# Emerging Trends in the Integration of Smart Sensor Technologies in Structural Health Monitoring: A Contemporary Perspective

**DOI:** 10.3390/s24248161

**Published:** 2024-12-21

**Authors:** Arvindan Sivasuriyan, Dhanasingh Sivalinga Vijayan, Parthiban Devarajan, Anna Stefańska, Saurav Dixit, Anna Podlasek, Wiktor Sitek, Eugeniusz Koda

**Affiliations:** 1 Institute of Civil Engineering, Warsaw University of Life Sciences—SGGW, 02-787 Warsaw, Poland; arvindan_sivasuriyan@sggw.edu.pl (A.S.); anna_podlasek@sggw.edu.pl (A.P.); wiktor_sitek@sggw.edu.pl (W.S.); eugeniusz_koda@sggw.edu.pl (E.K.); 2 Department of Civil Engineering, Aarupadai Veedu Institute of Technology, Vinayaka Missions Research Foundation, Paiyanoor, Chennai 603104, India; vijayan@avit.ac.in (D.S.V.); parthi92bhde@gmail.com (P.D.); 3 Division of Research and Development, Lovely Professional University, Phagwara 144411, India; sauravarambol@gmail.com; 4 Centre of Research Impact and Outcome, Chitkara University, Rajpura 140417, India

**Keywords:** automation, SHM, integration, monitoring, sensors, structural engineering

## Abstract

In recent years, civil engineering has increasingly embraced communication tools for automation, with sensors playing a pivotal role, especially in structural health monitoring (SHM). These sensors enable precise data acquisition, measuring parameters like force, displacement, and temperature and transmit data for timely interventions to prevent failures. This approach reduces reliance on manual inspections, offering more accurate outcomes. This review explores various sensor technologies in SHM, such as piezoelectric, fibre optic, force, MEMS devices, GPS, LVDT, electromechanical impedance techniques, Doppler effect, and piezoceramic sensors, focusing on advancements from 2019 to 2024. A bibliometric analysis of 1468 research articles from WOS and Scopus databases shows a significant increase in publications, from 15 in 2019 to 359 in 2023 and 52 in 2024 (and still counting). This analysis identifies emerging trends and applications in smart sensor integration in civil and structural health monitoring, enhancing safety and efficiency in infrastructure management.

## 1. Introduction

In recent years, the discipline of civil engineering has undergone profound changes worldwide, driven by a multitude of factors that vary across different regions. Across rapidly urbanising cities in Asia like Beijing, Shanghai, and Mumbai, civil engineering has been shaped by the imperative to accommodate expanding urban populations, leading to extensive infrastructure development such as skyscrapers, highways, and mass transit systems [1,2,3]. In contrast, in European cities such as Copenhagen, Amsterdam, and Stockholm, a strong emphasis has been placed on resilience and sustainability in response to climate change and environmental concerns. Projects here often focus on green infrastructure, renewable energy systems, and flood management initiatives to mitigate environmental impact. Meanwhile, in countries with advanced economies like the United States, Japan, and Germany, civil engineering has seen significant advancements in technological innovation, leveraging digital design tools, advanced materials, and Building Information Modelling (BIM) to enhance project efficiency and effectiveness [4,5]. Civil engineering efforts are concentrated on infrastructure rehabilitation and retrofitting to ensure safety and prolong infrastructure lifespan in regions grappling with ageing infrastructure, such as the United Kingdom, France, and Italy. As societies worldwide recognise the urgent need to address environmental concerns and optimise resource utilisation, civil engineers are increasingly tasked with designing, constructing, and maintaining infrastructure that minimises environmental impact while maximising efficiency and longevity [6,7]. Integral to these transformations is structural health monitoring (SHM), which has emerged as a cornerstone of modern infrastructure management practises. SHM involves the continuous or periodic monitoring of structural integrity, performance, and condition to ensure the safety, functionality, and durability of civil structures such as bridges, buildings, dams, and tunnels. By proactively assessing the health of structures, SHM enables engineers to detect and address potential issues before they escalate into costly failures or compromise public safety [8]. The integration of advanced technologies, brilliant sensor technologies, and automation has been instrumental in driving the evolution of SHM into a sophisticated discipline. Smart sensors with embedded electronics and wireless communication capabilities have revolutionised how civil engineers monitor and manage infrastructure assets. These sensors can collect real-time data on various structural parameters such as strain, vibration, temperature, and corrosion, providing engineers with unprecedented insights into the performance of civil structures. Furthermore, automation technologies have enabled the seamless integration of smart sensor data into decision-making processes, allowing for predictive maintenance and informed asset management strategies. By leveraging data analytics, machine learning, and artificial intelligence, engineers can analyse vast amounts of sensor data to identify patterns, anomalies, and potential risks, enabling timely interventions and optimised maintenance schedules [9,10].

The evolution of SHM represents a significant journey that has transformed how civil engineers approach infrastructure maintenance and management. It traces its roots back to the early 20th century when engineers first recognised the necessity of monitoring the performance of civil structures. Initially, SHM relied on rudimentary manual inspection techniques and basic instrumentation to assess structural integrity. However, as infrastructure projects grew in complexity and scale, it became evident that more sophisticated monitoring solutions were needed to provide timely and accurate data on structural health. Enter innovative sensor technologies, which have revolutionised the field of SHM beyond recognition [11,12,13]. Equipped with embedded electronics and wireless communication capabilities, smart sensors enable the continuous monitoring of structural parameters such as strain, vibration, temperature, and corrosion. Unlike traditional methods, smart sensors provide real-time data on structural performance, empowering engineers to detect potential issues early, accurately assess structural health, and implement targeted maintenance strategies before problems escalate [14]. Integrating smart sensors into civil infrastructure has become increasingly widespread, encompassing various applications and structures. From bridges and buildings to dams and tunnels, smart sensors are deployed to monitor the health and performance of critical infrastructure assets. These sensors not only facilitate the assessment of individual structures but also provide insights into broader trends and patterns that can inform asset management strategies at the network level. The knowledge of SHM has been used for some civil infrastructures for at least the last decade. Still, for the past decade or two, new computer-based systems have been created to assist with the needs of ageing infrastructure operators. The Aerospace Practice of SHM has proved reliable for many years. Even though the method is effective in civil engineering, the methodology is in progress in military applications.

**Figure 1:**
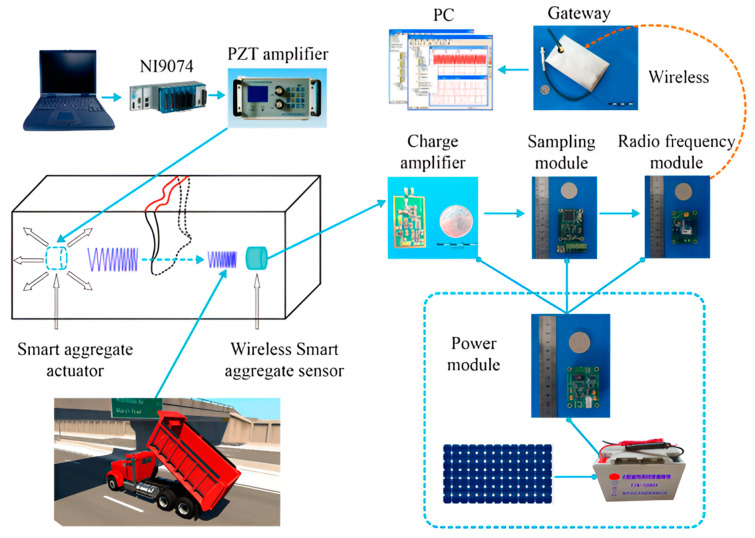
Depiction of the system setup and signal flow within the structural health monitoring (SHM) system. The components include PZT (lead zirconate titanate) sensors and a personal computer (PC). Adapted from Ref. [18].

The first step in implementing an SHM design is to specify the purpose of measurement. Discussing health and efficiency is crucial if we want a stable SHM framework. The sensor’s input data include operational and damage monitoring, while the sensor’s output data include damage identification and usage information [15,16,17]. The data collected from the sensors on the structures is utilised to verify and forecast the health of the structures. The output of this procedure is updated regularly based on the structure’s capacity to identify damage. In a civil engineering application, SHM detects damage and predicts the analysis of components in any structure. SHM observes and predicts structures through the order and consistency of periodically spaced observations, as shown in Figure 1.

The information on the current condition and future projection of the system’s health is based on extracting damage-sensitive choices from measured quantities using mathematical analysis. The monitoring system’s regular updates provide information on the structure’s power. It assesses the structure’s capabilities and capacity to forecast damage from standard operation. The widespread adoption of smart sensors in SHM has led to significant infrastructure maintenance and management advancements. Engineers now have access to a wealth of real-time data that enables them to make informed decisions, optimise maintenance schedules, and prolong the service life of infrastructure assets. Furthermore, monitoring structures continuously allows for the early detection of deterioration or performance deviations, thereby minimising the risk of costly failures and enhancing public safety.

**Figure 2:**
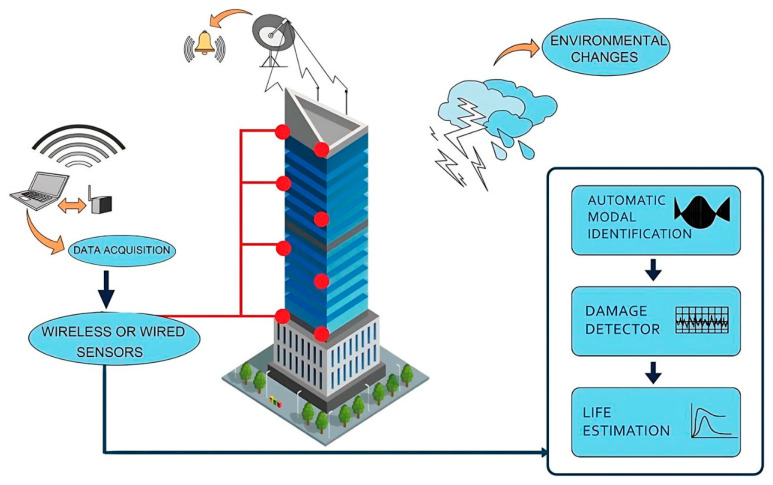
Sequence of SHM in multi-story buildings.

For instance, in an experiment, Zhang and Bai [19] demonstrated the utilisation of Radio Frequency Identification (RFID) broadcasting sensors in the field of engineering structures, using the Building Information Model (BIM) to scrutinise the distortion of the structure, which can predetermine deformation and identify the injury within time. Moreover, the BIM process monitored the whole circuit of the structure optically, and the Breakage-Triggered (BT) tension sensor, including the RFID label, scrutinised the damage situations of the structure in situations like ‘temblor’ without contact. Similarly, Almuhammadi et al. [20] investigated the actual-time dynamical blockade for the Carbon Fibre-Reinforced Polymer (CFRP) overlaid-exploded Quasi-Static Indentation (QSI) system in the structure to evaluate the hardly detectable interior deformation, which may lead to extreme injuries for the compound structure. In this experiment, two various electrode apparatuses were used in the position and orientation of the computations in the overlaid CFRP exterior factors for better pre-detection of damage. They reduced the destructive strength instantly after the completion of damage detection. Figure 2 depicts the workflow of SHM in multi-story buildings, representing sensor placement and environmental changes.

**Figure 3:**
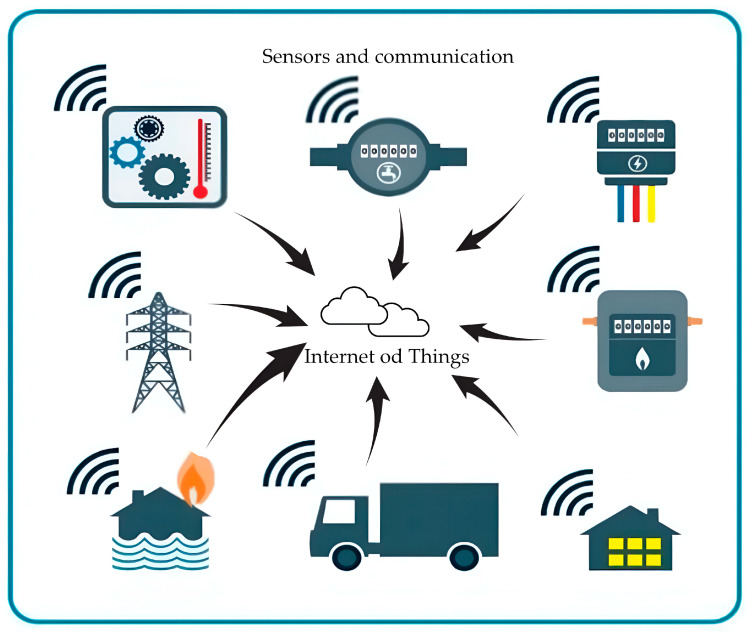
Depiction of the sensors and communication in various industries.

Similarly, Chacón and Zorilla [21] surveyed an analysis of a pragmatic, undeveloped 3D counterfeit of an additional cast iron bridge I-beam. This experiment progressively represented the analytical model and their statistics pre-locate the damage at the stage of construction and moulding of bridges. This survey aimed to negotiate pasture to prevent and monitor the actual-time structural deformation controlling readings and occurrences. Deng et al. [22] detected the pixel-levelled corrugated bridge structure based upon creativity by utilising the Atrous Spatial Pyramid Pooling (ASPP) assignment as a significant deface detector in the field of SHM. The structural bridge faced several problems, such as collecting the accurate adjunct of damage identification, oversizing the defacing area, and employing unskilled human resources for the bridge structure’s labelling. This ASPP network is used with two provocations. Moreover, the structure’s stable burden of Intersection over Union (IoU) deprivation must be considered to attain the same subdivision of volatile compact data files. Figure 3 depicts sensors and communication in different applications.

Dong et al. [23] expressed the techniques used for SHM, such as the XGBoost algorithm, to identify the concrete structure’s electrical resistance measurements. In this investigation, 16 record accreditations were collected, with 800 exclusive examples considered along the XGBoost algorithm to execute the outcome. Furthermore, the analytical Gridsearch CV and R2, Mean Absolute Error (MAE), and Root Mean Square Error (RMSE) methods were used alongside the RMSE measurements and elevated quantity of retrogradation installing line values for the statistical tests and training, with the cement content and preserving age having considerable impact on the outcomes of electrical resistivity measurement. Lin and Scherer [24] used horizontal counterfeits to represent a newly developed deface detector for concrete bridges. This detector uses the benefits of the extreme correctness of bridge reaction with debatable damage supposition for increasing deface detection capacity, decreasing the preparing period, and recording the dataset that fits the structural model using a Residual Sum of Squares (RSS) numerical process with differentiating to duplicate outcomes. Seven outlines are counterfeited for this proposed experiment. Ahmed et al. [25] inspected the fusion of carbon fibre, the unification of bucky tube recognising peel, and the constructional restoration of tiredness ruptured alloy structures, rectifying the process damage detection. Here, the discern coating unified in metal adherent is performed to observe the crack proliferation and distortion within the actual period and detect the ruptures using the conductivity substitute. The tiredness of life propagated from 380% to 500% due to the dependence on the conformation of the discerning coating. Zhu et al. [26] concluded the study on 4 × 4 detector assembling along a honeycomb sandwich combination to certify the health of the structural monitoring by suspecting Direct Coupling Mechanical Impedance (DCMI) and Comprehensive Active Monitoring Scheme (CAMS) impression. Moreover, the Root Mean Square Deviation (RMSD) and Damage Index (DI) are considered to evaluate and detect the not-bonded area innermost of the structure, where the functional Raw Signature (RS) is revived from the modified electromechanical impedance (EMI), which can be observed effectively.

McKenzie et al. [27] studied the Fibre Optical Sensors (FOSs) of connected reconstruction methodology for SHM. The defilement estimation strategies and contemporary Non-Destructive Inspection (NDI) are conducted in aircraft structures. Fibre Bragg Grating (FBG) sensors will survey the damage insemination under the reconstruction work procedure for this examination. This sensor utilised geographical and acumen composite strategies alongside Fabry–Pérot (FP) gauze to trace the independent matrix of damages. The capacity of these investigational outcomes for confirmed sensor recitation was used for the 3D finite element (FE) study and thermo-flexible scrutinisation. Wang et al. [28] monitored self-anticipating carbon epoxy compounds and nanofibers, their characteristics, and the operational procedures for SHM. The proposed carbon nanofibers (CNFs) or epoxy compounds were examined to improve the compounds’ hardness and Young’s modulus, and they conformed to the improved General Effective Media Equation (GEME). The value is 0.186 vol%. The outcome shows that adding carbon nanofibers based on epoxy compounds gives a new steady piezo resistivity and extreme vulnerability to enhance the workability efficiency of compressive strain sensors in engineering structures.

### Objectives of the Review

This review aims to provide a comprehensive overview of state-of-the-art smart sensor technologies for SHM. Specifically, it will:

- Survey the latest developments in innovative sensor technologies and their applications in SHM.
- Discuss the benefits and challenges of integrating smart sensors in SHM systems.
- Identify emerging trends and novel applications in SHM.
- Highlight opportunities for future research and collaboration to address key challenges and advance the state-of-the-art in SHM.

In light of these advancements, this review seeks to comprehensively explore state-of-the-art smart sensor technologies and their integration into SHM systems. The primary objective is to address key questions surrounding these technologies’ developments, applications, and performance in enhancing infrastructure safety and efficiency. Focusing on research conducted between 2019 and 2024, the review identifies novel applications, emerging trends, and future innovation and collaboration opportunities. By addressing the benefits and challenges associated with implementing smart sensors in SHM, this work aims to contribute to the evolving understanding and optimisation of monitoring practises for sustainable infrastructure management.

## 2. Methods

This approach involved a systematic analysis of the WOS Core Collection utilising particular keywords, including Automation, Health, Integration, Monitoring, Sensors, and Structural. The specified period of 2019–2024 delineates the period during which the dataset’s information was collected or published, signifying its relevance and contemporaneity [29]. The dataset draws from a broad array of 571 sources, encompassing journals, books, and potentially other publication types, indicative of its comprehensive coverage and diverse sourcing. With 1468 documents included, the dataset boasts a sizable volume of scholarly output, encompassing research articles, reviews, data papers, and more. Notably, the substantial annual growth rate of 28.23% underscores the dynamic nature of the dataset, reflecting increasing scholarly activity and interest in the subject matter over time.

Furthermore, the dataset’s average document age of 1.91 years highlights its focus on recent research, emphasising current developments and trends. The average citations per document, standing at 7.653, provide insight into the scholarly impact and relevance of the dataset, indicating the extent to which its contents have resonated within the research community. With data on 3987 authors, the dataset represents many contributors to the literature body covered. Notably, while most documents involve multiple authors, a small proportion of 79 papers are single-authored, indicating varied levels of collaboration. The dataset further illuminates collaboration patterns among authors, with an average of 4.52 co-authors per document and approximately 33.4% of documents featuring international co-authorships, highlighting global collaboration within the research community. Finally, the dataset encompasses various document types, including articles, data papers, and early access publications, offering researchers a comprehensive perspective on the research landscape within the specified timeframe.

### 2.1. Countries Production

**Figure 4:**
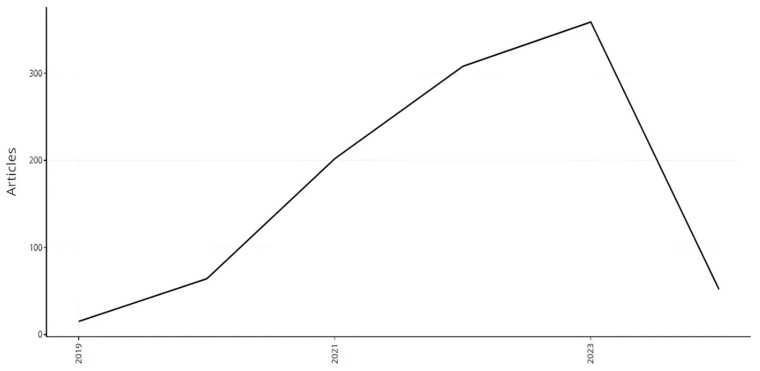
Illustration of the yearly scientific publications on integrating smart sensor technologies in SHM.

The annual scientific production shows (Figure 4) the number of articles published yearly from 2019 to 2024. The data reveals a clear trend of increasing scientific output over the specified period, with fluctuations in publication numbers observed from year to year. In 2019, scientific production commenced with 15 articles, indicating a modest start to the period under consideration. However, there was a notable surge in output in the subsequent year, with the number of articles published in 2020 more than quadrupling to 64. This substantial increase likely reflects growing research activity and interest within the field during that period. The growth trend continued in 2021, with a significant rise in scientific production to 202 articles. This marked acceleration suggests a continued momentum in research output, possibly driven by technological advancements, increased funding opportunities, or emerging areas of interest within the scientific community. The year 2022 witnessed a further escalation in scientific production, with the number of articles climbing to 308. This robust increase underscores the continued expansion of research efforts and the maturation of scientific endeavours within the field. In 2023, the upward trajectory in scientific output persisted, reaching 359 articles. This peak in production reflects sustained momentum and productivity within the research community, likely fuelled by ongoing advancements, collaborations, and knowledge dissemination efforts. However, the data also indicates a notable decline in scientific production in 2024, with the number of articles dropping to 52. While this decrease may seem significant compared to the previous year, it is essential to consider potential factors such as funding fluctuations, research priorities shifts, or external events that may have influenced publication trends during this period.

**Figure 5:**
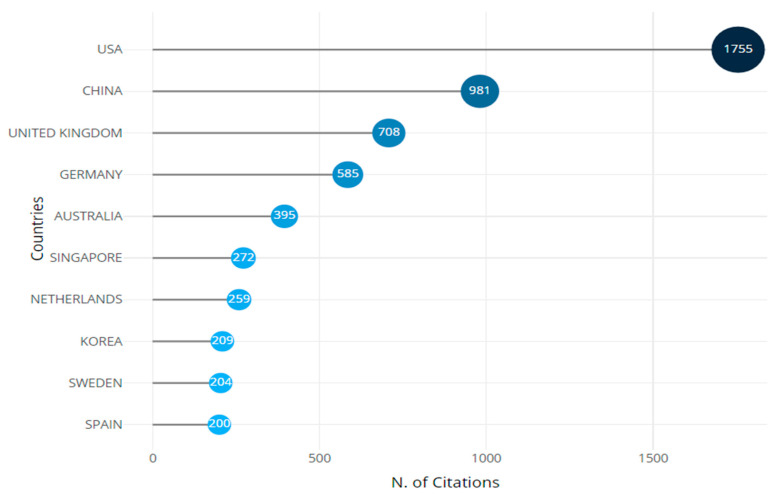
An example of the most frequently referenced nations is an article on integrating smart sensor technologies in SHM.

The trends of the most cited countries are identified in the context of international scholarly production, as depicted in Figure 5. The United States is the leading country with 1755 citations, although it has an average citation rate of 10.10. China comes second with 981 citations and the same average rate, which proves China’s dramatic increase in research and innovation. The United Kingdom occupies third place with 708 citations and an average rate of 7.50, which underlines the British tradition of scientific cooperation [30,31].

Germany stands fourth with 585 citations, and Australia occupies the fifth position with 395 citations. Both contribute to research with average citation rates of 5.50 and 7.50, respectively. Although Singapore has 272 citations and a 30.20 average citation rate per paper, it has published fewer papers than other countries, proving its high-quality research. The Netherlands, Korea, Sweden, and Spain constitute the rest of the top ten, and all exhibit a similar commitment to scientific quality, with citations varying between 204 and 259.

**Figure 6:**
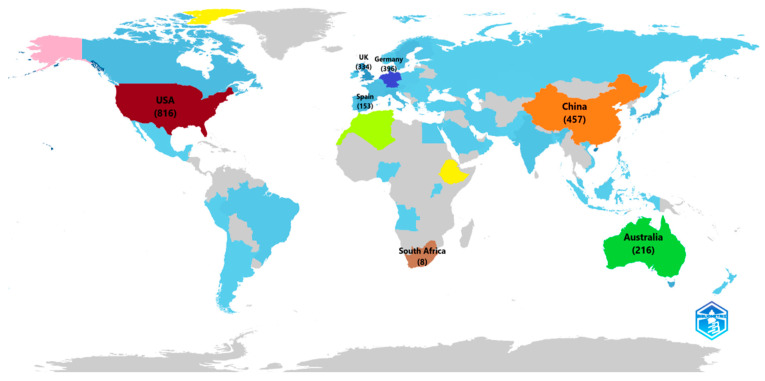
Illustration demonstrating the nation’s scientific output of papers on integrating smart sensor technologies in SHM.

Figure 6 shows the global research map based on the frequency of published articles. The United States tops the list with 816 publications, which indicates its advanced research system, creativity, and vibrant academic population. China comes next with 457 publications, which is justified by the country’s fast development and increased investments in research and innovation. The third place is occupied by Germany, with 396 publications. Germany is known for its engineering and technology and strengthens its position as a scientific European country. The leading country is the United Kingdom, which has 334 publications, explained by the presence of famous universities and research centres.

However, Australia has published 216 articles, less than the number of articles published in other countries, proving the country’s focus on science. The rest of the top ten are Spain, the Netherlands, Italy, Sweden, and Japan, which significantly invest in research and development and carve out their niches to solve social issues and stimulate the economy.

**Figure 7:**
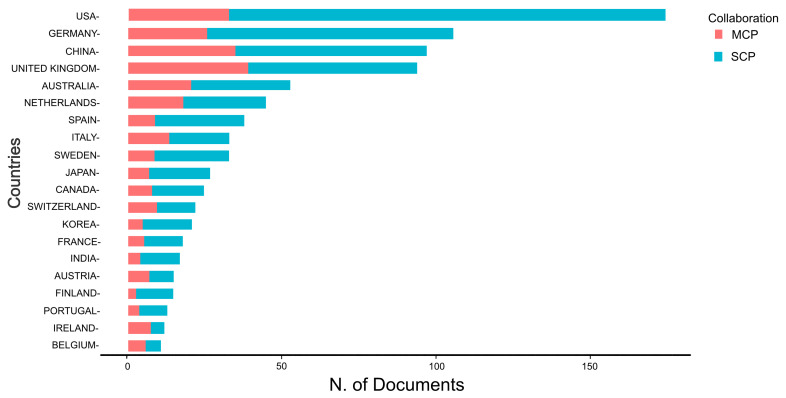
Illustration of the nations where the corresponding author researches integrating smart sensor technologies in SHM.

Figure 7 displays the corresponding authorship by country and the global research collaborations. The United States has the highest number of articles, with 174, of which 141 are single-country publications (SCPs) and 33 are multi-country publications (MCPs); the MCP ratio for the United States is, therefore, 0.190. Germany comes second with 106 articles, 80 of which are SCP and 26 MCP; the MCP ratio of which is higher at 0.245 due to increased collaboration with scholars from other countries. China comes third with 97 articles (62 SCP and 35 MCP), and the MCP/SCP ratio of 0.361 points to a concentration on international collaboration. The United Kingdom and Australia are next, with 94 and 53 articles, respectively. Both are involved in active international cooperation, which is reflected in the indicators of the MCP: 0.426 and 0.396. Other marked countries are as follows: the Netherlands, Spain, Italy, Sweden, and Japan also show similar research output and different extents of international cooperation, as indicated by their MCP ratios.

### 2.2. Most Frequent Words, Tree Maps, and Word Clouds

**Figure 8:**
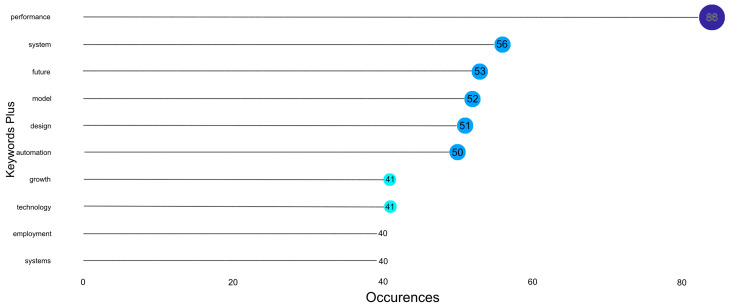
An illustration of the key terms from the publications on integrating smart sensor technologies in SHM.

The word “performance” is the most frequently used in the dataset, with a frequency of 86 occurrences, as shown in Figure 8. This is an essential point in that there is a significant emphasis on ‘measuring’ the performance of systems, technologies, or processes, with such key themes as efficiency. The term “system” comes next with fifty-six mentions, referring to different systems, technological and organisational systems, to be precise. The most frequently used word is “future”, which is used 53 and relates to trends, developments, and long-term opportunities. “Model” and “design” are used 52 and 51 times, respectively, underlining the significance of conceptual frameworks and system designs. Automate was used 50 times, and technology was used 41 times. They are included in essential discussions on technology and its application in processes and industries. Finally, ‘growth’ occurs 41 times and ‘employment’ 40 times, indicating the authors’ concern with economic growth, employment and the socio-economic effects of technological developments [32,33].

**Figure 9:**
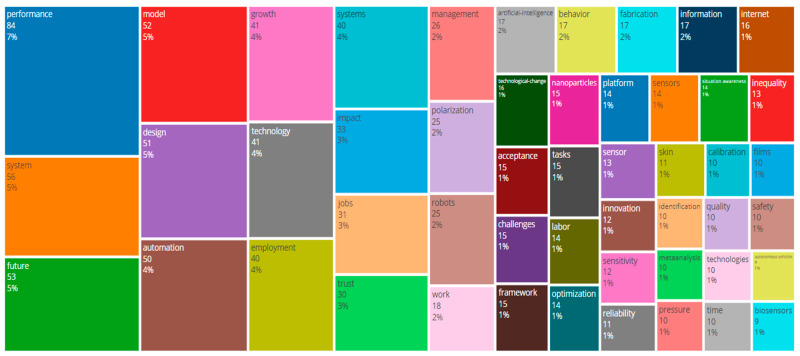
An example of how frequently the most pertinent terms are used in publications about integrating smart sensor technologies in SHM.

The TreeMap of the key terms in the dataset is given in Figure 9, and the size of the rectangle is proportional to the frequency of the term. ‘Performance’ is the most frequently used term, having occurred 86 times in the analysed content. After “performance”, words such as “system”, “future”, “model”, and “design” are also important, with frequencies of 51–56. The other smaller rectangles denote words like “automation”, appearing forty to fifty times, “growth”, “technology”, “employment”, and “systems”. The TreeMap allows users to quickly understand the primary terms and their significance in the context of the dataset by presenting a concise overview of the most often-used terms.

**Figure 10:**
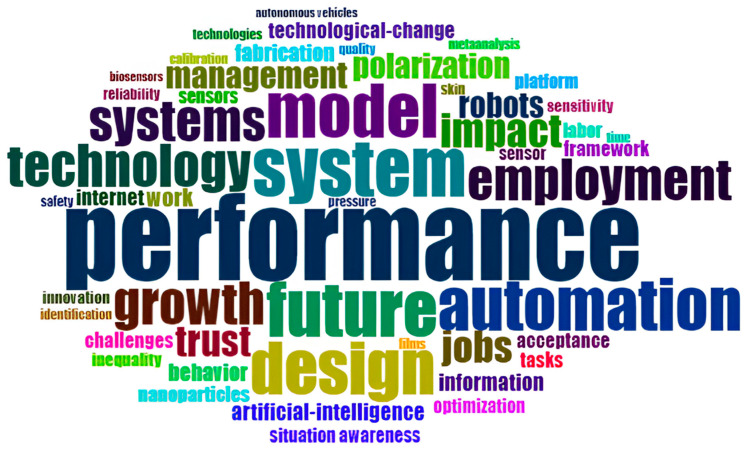
Illustration of the most pertinent keywords in bibliometric research on integrating smart sensor technologies in SHM.

In Figure 10, there is a WordCloud where the size of the word is proportional to the number of times it appears in the dataset. Among all the words, “performance” is the most frequent and is used 86 times to be discussed as the most significant and noticeable motif. Other terms such as ‘system’, ‘future’, ‘model’, ‘design’, ‘automation’, ‘growth’, ‘technology’, ‘employment’, and ‘systems’ are also identified with sizes that indicate their frequencies. The WordCloud is a fast and visually oriented view of the data, enabling the researcher to quickly find patterns, trends, and the most salient topics in the dataset.

A bibliometric analysis of the research on smart sensor technologies for SHM identifies global trends. The United States and China are also most productive in publication output and citation, which underlines their key position in developing this area based on the practical research base and international partnerships. Terms like ‘performance’, ‘system’, ‘future’, ‘model’ and ‘design’ underline the orientation towards improving SHM systems and analysis of its future trends. The TreeMap and WordCloud used in this study also support the need for performance evaluation, system design, and technological integration as key issues featured in the current literature. The presented results help us understand the changes in the research approach and the growth of interest worldwide in improving SHM systems. This is consistent with the paper’s goal of identifying new sensor technologies to increase the safety and efficiency of civil structures.

## 3. Results

### 3.1. Significance of SHM

SHM is a contemporary methodology that can replace conventional inspection by employing sophisticated sensors and data analysis to track structures continuously. SHM systems quantify strain, vibration, and temperature, among other factors, to give an overall picture of the condition of a structure. Through constant analyses of such data, SHM can identify the precursors to failure and act on them before the structure succumbs to them, thus increasing its durability [34,35].

Further, in case of an earthquake or explosion, SHM provides real-time feedback. This allows engineers to rapidly evaluate the structural condition and damage, where repairs should be made first, and if the structure is safe for the occupants and nearby property [36,37]. Further, SHM assists in predictive maintenance by constantly monitoring the health status of a structure to identify emerging patterns indicative of problems. This proactive approach allows engineers to schedule maintenance activities effectively and to use resources to the maximum while incurring minimum time off and disruptions. It also has a vital role to play in post-event analysis. Engineers can learn how structures perform under pressure by studying information collected during an earthquake. The insights gained from such analysis are beneficial in future design, retrofit, and risk management of civil structures, thereby increasing their resilience. Ji et al. [38] investigated the characteristics of lead-free piezoelectric nanofiber modules under different conditions. The study showed that poled modules improve the monitoring features with the help of XRD and dielectric measurements. This result highlights their usefulness for SHM applications in accurately evaluating structural conditions and detecting framework problems.

Huang et al. (2024) introduce a novel framework to reduce the few-shot data-driven structural damage identification (SDI) methods. They employ ASAPSO-CNN and data enhancement techniques to improve the performance of SDI. The process is tested on an experimental model of a three-span continuous beam and compared with four other data-driven methods, which show that it is effective and performs well under limited data conditions [39].

### 3.2. Need of Sensors in SHM

SHM constantly expands its sensor and sensing network portfolio, leveraging commercially available and proven sensors, signal conditioning, and data acquisition systems based on the individual sensor and data acquisition components’ physical, electrical, and thermodynamic properties in various applications [40,41]. Sensor resilience in harsh environmental conditions and reliable data transmission are paramount in civil infrastructure applications. Sensors must withstand extreme temperatures, humidity, vibrations, and exposure to corrosive substances without compromising performance. Low power consumption is crucial in remote or inaccessible locations to ensure long-term operation and minimise maintenance requirements. To ensure the effectiveness of SHM systems, careful planning of installation requirements for each sensor and data acquisition component is essential. This involves sensor placement, mounting methods, calibration procedures, and power supply arrangements [42,43,44,45].

Ren et al. [46] stated that a simple, rapid, and sensitive radiometric fluorescence sensor was developed to determine the scene partiality of trace resorcinol (RS) in its isomers and proportions; the unique coupling response between RS and DA (dopamine) produces fluorescent azamonardine (AZMON) within two minutes, and the flu fluorescence rapidity increases with the RS concentration. The growing heed of RS set off the explicit variation in fluorescent colour. From red to blue, rooted on the introductory red fluorescence of AUNcs (Au nanoclusters) and RS was 5.0 nm, which can be recognised with a stripped eye. The variation in fluorescent colour as a substitute for fluorescent effulgence is specifically suitable for visual invigilation. The introduced sensor has been successfully used to fix RS in actual water patterns and is comfortable for tracing RS’s routine dissection and field invigilation.

Hegedűs and Czigány [47] aim to indicate that the single-mode ocular glass fibre in applied science and the ocular damage trial set used to portray information technology networks are preferable for the SHM of polymer agglutinates. Founded on the outcomes, it worked out the fundamentals of the economic metering process. They constructed optical fibres into patterns and scrutinised the connection amidst the fibre depletion and the instance’s defacement. The system can be applied in training, estimated lightly, and exhibits a rigorous anatomical examination. Similarly, Yang discussed that multiple broadcaster fibre optic dislocation bale sensors have been investigated for extensive amplitude oscillation metering. Delegating various transmitters, Oyadiji [48] expressed that a fixed ratio in the first and second layers improved the linear limit and output sensibility. Both pretension and realistic results advocate for a linear performance cap of approximately 3 mm, nearly four times that of the conventional bisection design. The outcomes exhibited a relevant sensitivity of about 0.36 mv/μm of bandwidth up to 2.2 KHZ with a dislocation amplitude of about 50 nm and indication-to-sound ratio on all sides of 26 GB. A harmonic stimulation trial is deputed to conclude the dynamic dislocation reaction dealings of the sensor. Biondi et al. [49] presented the foundation for a first-time display of humidity sensors rooted on low calcium fly ash geopolymers, a class of cementitious, and electrolytically portative remake fixings. Electrochemical hindrance spectroscopy and coordinate circuit models are applied to design geopolymer sensors’ electrical reactions to water saturation and temperature. Also, sensor reactions within 3% amidst wetting and caloric cycles occurred last. This experiment’s outcomes showed spectrometry and chromatography explanations for the least ion leaching from the sensors. This study is expected to be the first phase in evolving 2D dispensed sensor remakes for solid frameworks and other chemical sensors that help solid structural health monitoring and warnings.

### 3.3. Fibre Optic Sensors (FOS)

Fibre optic sensors (FOS) are now widely recognised as an efficient and effective means of SHM. They are ideal for real-time monitoring of the structure’s health since they can measure parameters such as strain, temperature, vibration, and displacement [50,51]. FOS has essential benefits because of its high sensitivity, immunity to electromagnetic interference, and the possibility of local and global monitoring. For instance, Zheng et al. [52] discussed the development of FOS technologies, compared bend-loss-based sensors with Bragg grating sensors for geotechnical applications, and established their capability to convert strain signals into useful information.

The localised application of FOS helps engineers place sensors directly into the structural members where early signs of strain or temperature changes are likely to occur so that maintenance can be targeted and large-scale failure avoided [53,54]. On a larger scale, FOS facilitate the system-level performance monitoring of structures like bridges, dams, and pipelines and provides long-term trends [55]. For example, Gianti et al. [54] used FOS in bridge vibration measurement, while Sasi et al. [56] proved FOS in railway structures to avoid expensive repair by detecting initial signs of damage. 

**Figure 11:**
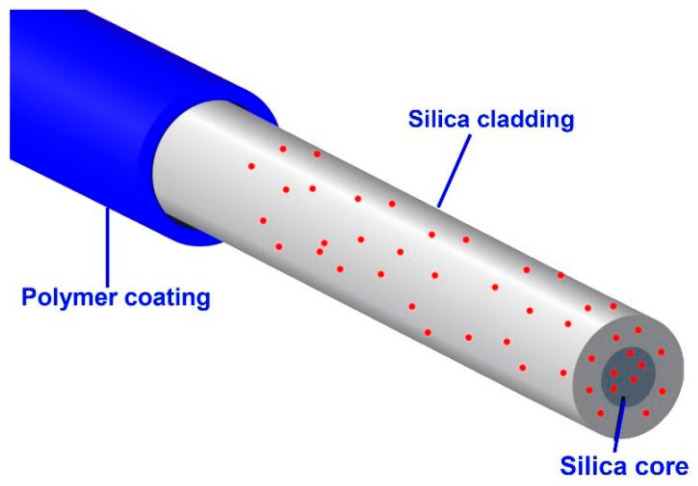
Fibre optic sensor for SHM application.

Furthermore, FOS is improving with developments such as distributed sensing technologies and coupling with FE models for accurate thermal and deformation estimation of concrete slabs [57,58]. Newer technologies like FOSS and new coatings for reinforcements take their uses to crack detection, corrosion, and material degradation assessment [59,60,61]. Figure 11 shows FOS for SHM purposes.

FOS have been highly beneficial for SHM because of their precision, the ability to withstand several years of service and resistance to EMI. Due to their capacity to track damages at the local level and the overall performance, they are preferred for essential structures. However, challenges like cost and the necessity of high-level data processing are still present. Further studies should aim to increase FOS cost-effectiveness and the compatibility of FOS with innovative technologies such as big data analytics for predictive maintenance.

### 3.4. Piezoelectric Sensors

Piezoelectric materials are renowned for their unique ability to convert mechanical energy into electrical energy and vice versa through a phenomenon known as the piezoelectric effect. This remarkable property makes them highly suitable for sensor applications where the precise measurement of mechanical parameters is required. Piezoelectric ceramic sensors have recently emerged as a prominent choice for structural health monitoring (SHM) in civil engineering systems [62,63]. Piezoelectric ceramic sensors utilise electrical impedance and elastic wave measurements to monitor the health and integrity of civil engineering structures. These sensors detect mechanical vibrations, strains, and deformations within the monitored structure. When subjected to mechanical stimuli, such as stress or vibration, piezoelectric ceramic sensors generate electrical signals proportional to the applied mechanical force. Integrating piezoelectric ceramic sensors into SHM systems offers several advantages [64,65]. Firstly, their high sensitivity and responsiveness enable the detection of subtle changes in structural behaviour, allowing for the early identification of potential issues or defects. Additionally, piezoelectric ceramic sensors can operate in harsh environmental conditions, making them suitable for long-term monitoring applications in civil engineering. Furthermore, piezoelectric ceramic sensors can be deployed in active and passive sensing configurations. An external excitation signal is applied to the sensor in active sensing, and the resulting electrical response is analysed to assess structural health. Passive sensing involves monitoring the natural vibrations and reactions of the structure to environmental stimuli without external excitation. This dual capability provides flexibility in monitoring different structures and operating conditions. Bhalla et al. [66] studied a lengthy practical work linked to low-strain weariness loss, controlling the addition of a real-life-sized RC fabrication. Concrete vibration sensors (CVS) control the dual mode, acting as sensors for universal vibration technology and regional EMI technology. The fixation of the framework’s overall rigidity by the similar CVS supplied alternate loss measures in the precondition of an original calculation of the remaining flexural rigidity. Hasni et al. [67] expressed using self-powered piezo-floating (PFG) sensors with alterable injection rates as the latest anatomical health monitoring process. Various features were extracted from the agglomerative voltage down for every memory gate rooted on the sensor group concept to enhance the loss find precision. An optimum method evolved to optimise the classifier’s parameters to improve the finding rate precision. The execution of the introduced system is satisfactory for finding loss progression in steel sheets.

Moharana and Bhalla [68] studied the re-derived duet entrance sign rooted in the flowing transform of dislocation and charges over the piezoelectric Lead Zirconate Titanate (PZT) patch. It removes the brother of fitment, the coordinate impedance of the framework, and the effector differently. The outcomes are proportioned with the old models to point to the superior precision of the new perspective. A continuum-rooted interplay has been derived from the latest model to quantify the clipper’s lag and inertia impacts—similarly, Zhang et al. [69] described the civil engineering remake patches as improving competency and functionalities. The embedded sensors were utilised to control cold treatment for agglutinate particles and evaluate present monitoring technology for bio-sourced fibre-strengthening composites. Embedded framework health monitoring in coalescent material with hindrance testing is a multipurpose process controlling agglutination at various life stages.

**Figure 12:**
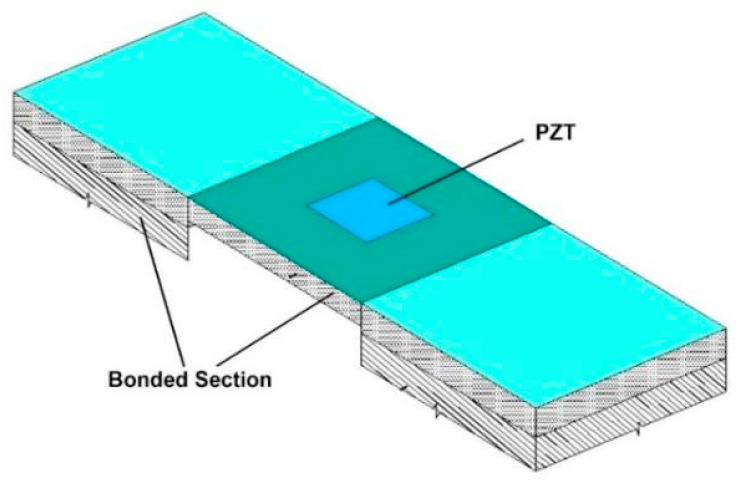
Piezoceramic sensors in concrete.

Haq et al. [70] investigated the possible application of EMI technology for diagnosing superior cycle and strain weariness loss in reinforced concrete (RC) frameworks. The loss incumbent equivalent stiffness parameter (ESP) has occurred from the conductivity and the susceptance signatures for every concrete vibration sensor (CVS) at various loss states for active calculation of remaining stress life. A connection for calculating the framework’s remaining stress life with the change in simple maceration has been derived for the loading atmosphere. Additionally, Haq et al. [71] presented a fictional SHM perspective rooted in the wavelet power of entrance signals to find, regionalise, and calculate the severity of the losses caused by stress in strengthening concrete frames. The intention of calculating wavelet powers Discrete Wavelet Transformation (DWT), Continuous Wavelet Transformation (CWT), and Power Spectral Density (PSD) explication is that they are used on actual entrance signals in frequency estates. The practical outcomes certify the higher exhibition of the DWT-rooted optimal procedure in activating the RC framework’s full-fledged exact time loss oracle beneath low strain and superior cycle stress [72,73,74,75,76]. Figure 12 shows the implementation of SHM in a concrete structural element using Piezoceramic sensors.

### 3.5. Measuring Acceleration

Accelerometers are electromechanical devices designed to measure acceleration forces experienced by an object along one or more axes [77,78,79,80]. These forces can be either static or dynamic. Static forces refer to constant accelerations, such as the gravitational force acting on structural elements. In contrast, dynamic forces involve varying accelerations, such as those experienced when a vehicle moves or machinery operates [81,82,83,84]. In civil engineering, accelerometers are crucial in monitoring the dynamic state of structures and infrastructure. They measure vibrations, shocks, and movements within buildings, bridges, dams, and other civil engineering projects. Accelerometers provide valuable insights into infrastructure assets’ structural health and performance by accurately detecting and quantifying these accelerations. One of the key applications of accelerometers in civil engineering is structural health monitoring (SHM). By continuously monitoring accelerations along different axes, accelerometers can detect abnormal vibrations or movements that may indicate structural defects, deterioration, or damage. This early warning system enables engineers to assess the integrity of a structure, identify potential hazards, and implement timely maintenance or repair measures to prevent structural failure. Accelerometers are also used in the design and testing of civil infrastructure projects. They help engineers evaluate the dynamic response of structures to various loads, including wind, seismic activity, and vehicular traffic. By simulating these external forces and measuring the resulting accelerations, engineers can optimise the design and ensure infrastructure projects’ structural integrity and safety [85,86].

Furthermore, accelerometers find applications in monitoring construction activities, such as pile driving, tunnelling, and excavation. They enable engineers to assess the impact of construction activities on nearby structures and underground utilities, ensuring compliance with safety regulations and minimising the risk of damage. In addition to civil engineering, accelerometers are widely used in various fields, including aerospace, automotive, healthcare, and consumer electronics. They are integrated into mobile phones, gaming consoles, fitness trackers, and industrial machinery to measure acceleration, detect motion, and provide orientation information.

### 3.6. Measuring Forces

**Figure 13:**
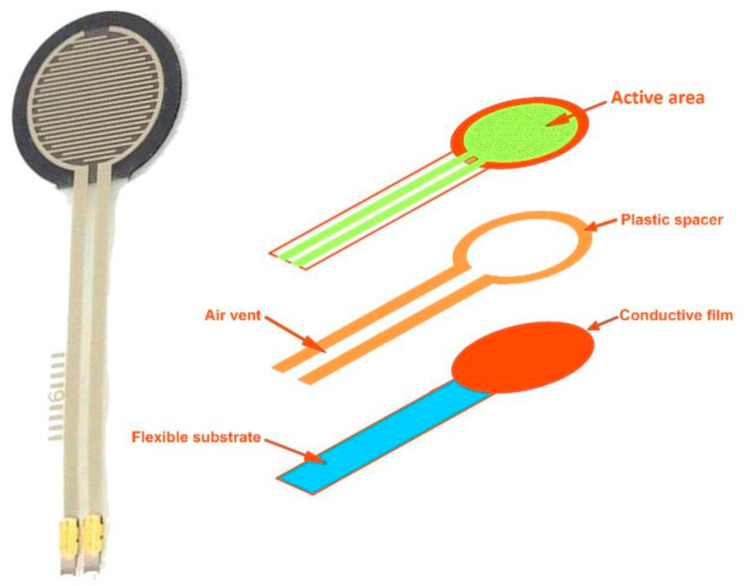
Force sensors and their components for SHM application.

Force sensors are essential components used in structural monitoring to detect and quantify external forces acting on civil infrastructure. These sensors produce a measurable reaction to the force applied to the structure, providing valuable information about the structural response to external loads or disturbances [87,88]. The operation of force sensors relies on various principles of force measurement, including strain gauge, piezoelectric, capacitive, and electromagnetic induction. Each type of force sensor has a unique mechanism for detecting and quantifying forces, allowing for flexibility in monitoring different structures and environments. In civil engineering applications, force sensors are critical in assessing the structural integrity and performance of bridges, buildings, dams, and other infrastructure assets [89,90]. By strategically installing force sensors at key locations within the structure, engineers can monitor the distribution and magnitude of forces exerted on critical components, such as beams, columns, and foundations. The data collected by force sensors enables engineers to analyse the structural response to various loading conditions, including static loads (e.g., gravitational forces) and dynamic loads (e.g., traffic loads, wind loads, seismic forces). By monitoring changes in force levels over time, engineers can identify potential issues such as overloading, fatigue, or structural degradation, allowing for timely intervention and maintenance. Figure 13 displays force sensors and their components for SHM practises.

### 3.7. Microelectromechanical (MEMS) Devices

Micro-electromechanical systems (MEMS) technology has revolutionised structural health monitoring (SHM) by offering compact, efficient, and cost-effective solutions for predicting and measuring vibrations in civil structures [91,92,93]. MEMS devices consist of tiny mechanical and electrical components integrated into a single microchip, allowing for precise sensing and analysis of structural vibrations. One of the key advantages of MEMS technology in SHM is its ability to predict and detect increases in structural vibration levels [94,95,96,97]. By measuring accelerations, strains, or displacements with high sensitivity and accuracy, MEMS sensors can identify changes in the dynamic state of a structure, which may indicate structural damage, deterioration, or excessive loading. This predictive capability enables engineers to proactively assess the health and integrity of civil structures, allowing for timely intervention and maintenance to prevent potential failures or hazards [98,99,100].

Moreover, MEMS devices are known for their compact and lightweight design, making them highly suitable for integration into structural monitoring systems. Their small size allows easy installation in hard-to-reach or confined spaces within a structure, enabling comprehensive monitoring of critical components and areas. Additionally, MEMS sensors consume minimal power, making them ideal for long-term, autonomous monitoring applications requiring continuous operation.

**Figure 14:**
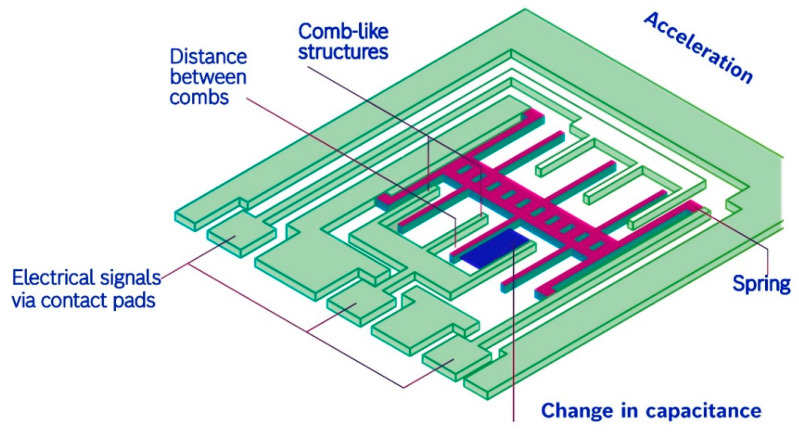
MEMS for acceleration monitoring.

Furthermore, MEMS technology offers superior performance and reliability compared to traditional sensing methods. These devices are engineered to withstand harsh environmental conditions, such as temperature variations, moisture, and mechanical shock, ensuring accurate and dependable operation in real-world applications [101]. Additionally, MEMS sensors can provide high-resolution data with fast response times, enabling engineers to capture detailed information about structural vibrations and dynamic behaviour. Tondolo et al. [102] demonstrated that this research establishes a new paradigm for instrumented reinforced steel bars incorporating traditional low-cost embedded MEMS strain sensing capabilities. There is a requirement for a more cost-effective operating process in the civil framework and infrastructure. A low-cost embedded process was used to build the concrete structure. Multiple trials are reported to elaborate on the ability of the method. Practice up to compress demonstrates a considerable field of appropriation. Guidorzi et al. [103] noted that some families of more variable quantities models can be applied in SHM-oriented recognisance systems, especially the extension of AR models, to ponder the existence of extra-size noise. A high-scale analogy is described amidst these models’ strength spectral densities and those of the data applied for their recognition. The outcomes are given by the contemplate systems and a juxtaposition of the execution of the MEMS-rooted process with a conventional solution rooted in piezoelectric seismic accelerometers [104]. Figure 14 displays MEMS sensors to monitor acceleration.

### 3.8. Measuring Displacement

Monitoring displacement in structures is crucial due to its direct correlation with structural integrity and potential failure [105,106]. Excessive displacement can indicate structural issues such as overloading, foundation instability, or material degradation, making accurate measurement essential for early detection and timely intervention. In structural health monitoring (SHM), two standard technologies used for displacement sensing are Global Positioning Satellites (GPS) and linear variable differential transformers (LVDTs). GPS technology offers global coverage and high accuracy in measuring absolute displacement over large areas, making it suitable for tracking large-scale movements in structures like bridges or dams. However, GPS may be limited by signal obstructions or atmospheric conditions in urban or vegetated areas. On the other hand, LVDTs provide high resolution, accuracy, and repeatability in measuring displacement, making them ideal for monitoring small-scale movements or deformations in structures such as buildings or pipelines. However, LVDTs require physical contact with the structure, which may not be feasible in all applications.

### 3.9. Global Positioning Satellites (GPS)

In the last twenty years, GPS geodetic-grade receivers and accelerometers have emerged as essential tools in structural health monitoring (SHM) to measure civil structures’ static and dynamic conditions. GPS technology in particular offers near-real-time accuracy, ranging from millimetres to a few centimetres, thus suitable for capturing incremental structural movements of bridges, buildings, and dams [107]. Integration of GPS receivers and accelerometer networks allows engineers to use displacement GPS and vibration data accelerometer to obtain a complete picture of the structure’s performance.

Kim et al. [108] have used the RTK-GPS sensors for displacement monitoring of Yeongjong Grand Bridge and achieved a precision rate of 2 mm at 100 Hz, proving the effectiveness of modern GPS sensors. Similarly, Vazquez et al. [109] employed GPS technology to assess the 45-year-old Juarez Bridge and detected critical displacements and structural problems through permanent monitoring and sophisticated filtering analysis.

When integrated with accelerometers, GPS provides the most accurate measure of static displacement and dynamic force for early identification of structural changes. This dual capability helps carry out preventive maintenance, hence minimising failure cases. However, problems like signal obstructions, environmental interference, and the need for more sophisticated filtering algorithms persist. Subsequent developments should increase GPS precision in urban or obstructed environments and incorporate AI analysis of GPS and accelerometer data for real-time interpretation to make SHM systems more effective and dependable. 

### 3.10. Linear Variable Differential Transformer (LVDT)

**Figure 15:**
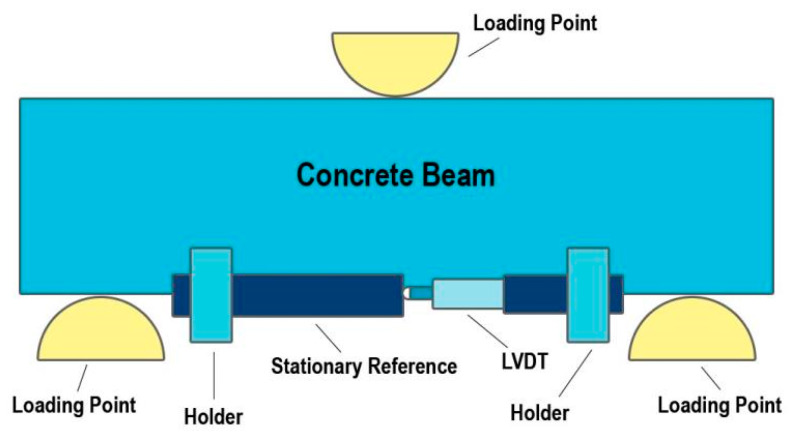
Concrete beam experiment using LVDT.

The linear variable differential transformer (LVDT) is a widely used type of displacement transducer renowned for its accuracy, reliability, and versatility in measuring linear displacement. Its design comprises a hollow cylindrical shaft containing a solid cylindrical core and a series of inductors [110,111,112]. The core is typically made of ferromagnetic material, allowing it to interact with the electromagnetic fields generated by the inductors. In the case of the LVDT, the primary and secondary coils are located on opposite sides of the primary core. When the core is shifted from the centre of the hollow cylinder to another position, the mutual inductance of the secondary and primary coils varies. This variation in mutual inductance is directly proportional to the displacement of the core within the cylinder. The displacement of the core changes the magnetic field that links the primary and secondary windings of the LVDT and hence changes the output voltage [113,114]. This output voltage is linearly related to the core’s position within the cylinder, allowing for precise measurement of displacement over a wide range. One of the key advantages of LVDTs is their ability to provide accurate and repeatable measurements, even in harsh environmental conditions or high-vibration environments. Additionally, LVDTs are highly durable and resistant to wear and tear, making them suitable for long-term monitoring applications in various industries, including aerospace, automotive, and civil engineering. One team of researchers presented the bridge during trials of the anatomical credibility of a pre-stressed concrete bridge made in the late 1960s. The execution and rates of jointly directed static and dynamic load were trialled. A sophisticated finite element model of the bridge was created because of the dynamic load trial. The comparison demonstrated that emotional load trials could be used in static load trials for anatomical testing of new bridges or to monitor operational bridges. Tung Khuc et al. [115] expressed that the new conviction of vision-rooted dislocation metering applying an Unmanned Aerial Vehicle (UAV) introduced in the work exhibited the potential to impassively measure the dynamic dislocation of tower framework in time series. Noncontact vision-based metering technology is currently granted as a primarily thinkable perspective, albeit some limitations still mark it. The translations created by the UAV were acquired employing context in the background. The introduced process was certified practical with a small-sized steel tower, and the results have given a starting adaptation of the prospective promising eventuality. Figure 15 depicts the experimental method for concrete beams using LVDT.

Baas et al. [116] reported that a methodological perspective to managing mass wood commercials’ operating data was introduced. The stand is certified, and ten months of static and hygrothermal operating data are applied. The data explains the moisture performance of top panels and problem damage in post-tensioned shear walls. SHM is used to calculate new anatomical methods and critical construction. Additionally, Havaran and Mahmoudi [117] presented a new algorithm to track line points connected to Article 40 to remove dynamic anatomical competencies, such as mode sizes and original frequencies. The randomised Hough finds algorithm is applied to perceive the rectangle markers’ movement on the framework. The outcomes have been juxtaposed with measurement technology, such as the Lucas Kanade algorithm, finite element process, and accelerometer sensors. The results show that the new algorithm enhances computer vision in verifying points for taking out dynamic anatomical competency.

### 3.11. Measuring Velocity

Velocity sensors play a crucial role in SHM by capturing the movement of structures and providing valuable data for predicting potential damage [118,119]. These sensors utilise two main sensing methods: electromechanical impedance techniques and the Doppler effect, each offering unique capabilities in measuring structural velocity. Electromechanical impedance (EMI) techniques involve monitoring changes in the electrical impedance of a structure caused by mechanical vibrations. This method typically employs piezoelectric materials or sensors attached to the structure’s surface. As the structure vibrates, the impedance of the piezoelectric material changes, allowing for the measurement of velocity based on the rate of change in impedance. EMI techniques offer high sensitivity and are well suited for detecting subtle structural movements and vibrations.

On the other hand, the Doppler effect is utilised in velocity sensors by measuring the frequency shift in reflected waves caused by the motion of the structure. This method involves emitting a wave, such as ultrasound or laser, towards the structure and measuring the frequency shift in the reflected wave due to the motion of the structure [120,121]. The magnitude of the frequency shift is proportional to the velocity of the structure, allowing for accurate velocity measurement. In recent years, SHM has increasingly adopted velocity sensors because they capture structural movement with precision and accuracy. By continuously monitoring the velocity of structures, engineers can identify changes in dynamic state that may indicate potential damage or deterioration. This predictive capability enables proactive maintenance and intervention to mitigate risks and ensure the safety and integrity of civil infrastructure.

### 3.12. Electromechanical Impedance (EMI) Techniques

The electromechanical impedance (EMI) technique successfully identifies structural damage by employing a high-frequency voltage source to stimulate localised zones and record alterations in impedance. Changes in impedance describe changes in the mechanical characteristics of a structure and are good indicators of damage, degradation, or irregularities [122,123]. Due to this high sensitivity, EMI is valid when used at the initial stages of damage, where early repair prevents further deterioration in structures such as buildings, bridges, and pipelines.

**Figure 16:**
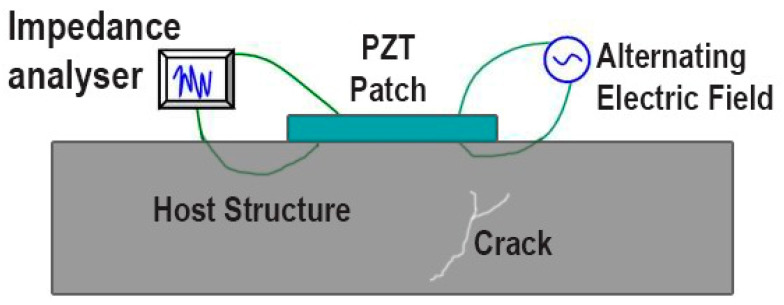
EMI techniques to measure cracks in beams.

The supplement of frequency ultrasound analysis with EMI enables engineers to describe the structure’s state based on the system’s dynamic frequency response. For instance, Silva et al. [124] have shown the possibility of using EMI-based PZT sensors to characterise the fragility of Duplex Stainless Steels (DSS) by detecting changes in structural stiffness under thermal conditions. Likewise, Wang et al. [125] designed a piezoelectric EMI sensor to detect bearing wear, demonstrating how changes in wall thickness affect wear performance. These studies also emphasise that EMI can accurately pinpoint even the slightest modifications in the structure of a building. Figure 16 represents the experimental setup by adopting EMI techniques to measure cracks in concrete beams.

Li et al. [126] presented a combined Electromechanical Admittance (EMA) or electromechanical impedance (EMI) strategy for constructional damages in different temperatures with Convolutional Neural Network (CNN) to evaluate the Pearson Correlation Coefficient (PCC) statics. Using the EMA strategy, these CNN data were substantiated to assess the precise damage amount and temperature identification using the Orthogonal Matching Pursuit (OMP) technique. Li et al. [127] experimented with an OMP for EMA of SHM along reconversion and signal compaction perspective by utilising a PZT actuator to detect local destructions in various situations. The improved OMP was discussed with some algorithms for supported beams according to their perceptible and categorical data. This whole procedure gives optimum compensation to EMA/EMI based on the structure of the SHM convention.

The strength of the EMI technique is that it is susceptible and can identify early signs of damage, especially in complex and localised components. EMI is instrumental in making infrastructure management cost-effective by providing means to maintain only the necessary areas and avoid extensive failures. However, there are several limitations, including the determination of the optimal location of the sensors, the problem of noise, and the fact that more elaborate signal processing algorithms are required to increase the precision of the readings. Possible developments for the future of EMI could be its combination with artificial intelligence data analysis and multiple sensors to increase the speed and accuracy of damage detection and monitoring in various structures.

### 3.13. Doppler Effect

Vibration measurements have a rich history in structural health monitoring (SHM), with various techniques and technologies employed to assess structures’ dynamic state. Laser-Doppler vibrometers have been a prominent choice for measuring mechanical vibrations in SHM applications [128,129,130]. These instruments operate by directing laser beams onto the surface of a structure and measuring the Doppler shift in the reflected light, allowing for precise measurement of surface vibrations. In addition to Laser-Doppler vibrometers, radar interferometry has emerged as an alternative method for detecting vibrations in large buildings and structures. This remote sensing technology utilises radar waves to detect slight deformations across long distances. The operating principle of radar interferometry involves measuring phase variations in reflected electromagnetic radiation using two antennas positioned close to each other. By analysing the phase differences between the received signals, engineers can accurately quantify the magnitude and frequency of structural vibrations. One of the key advantages of radar interferometry is its ability to monitor structural vibrations from a remote location without direct contact with the structure [131,132]. This capability makes it particularly useful for monitoring large-scale structures such as bridges, towers, and high-rise buildings, where traditional measurement techniques may be impractical or challenging to implement.

**Figure 17:**
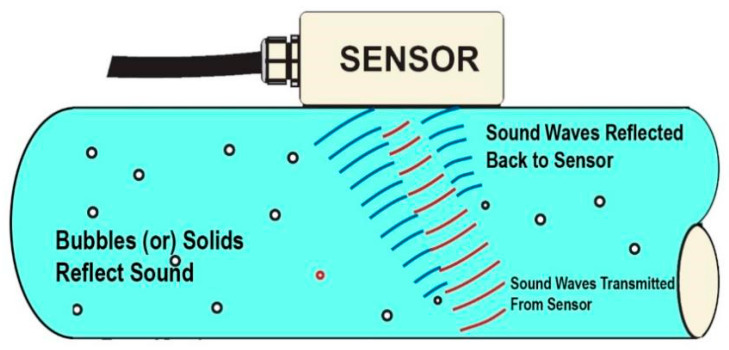
Doppler effect techniques in SHM.

Additionally, radar interferometry offers high sensitivity and resolution, allowing for the detection of subtle vibrations and deformations that may indicate structural issues or damage. Furthermore, radar interferometry enables continuous monitoring of structural dynamics over extended periods, providing valuable insights into civil infrastructure’s long-term performance. By capturing data on structural vibrations, engineers can assess the health and integrity of structures, identify potential risks or vulnerabilities, and implement targeted maintenance and repair strategies to mitigate these risks. Dong et al. [133] represented the recognition of vigorous constructional properties based on machine vision sensing automation. The vigorous infrastructural displacement was measured simultaneously for the multi-point computation process to observe these properties. Based on the vision-based procedure, a steel rectangular supported beam was adopted to survey computation outcomes’ practicability, impact, and differentiation. At last, this method was considered more exact, impactful, and steady for modal specification recognition and vigorous structural reaction monitoring. Figure 17 displays the process of the Doppler effect in SHM.

Rothberg et al. [134] discussed how the Laser Doppler Vibrometer (LDV) was introduced in 1964 at Columbia University through various investigations after the invention of the laser. This investigation showed the advancement of various LDV strategies soon after the recurrence transfer of the scrambled light experiment was made. Importance was given to the provocation faced due to Laser flecks. However, this experimental replica introduced many influential experiments based on MEMS and SHM in various sizes. Many implementations of acoustics and classics are shown in the province of sounds and anticipation by exploring hearing and Rotor Vibrations. In conclusion, this field has a great future that can be analysed with modern tools.

Linzer et al. [135] aimed to find optimal designs for single axle sensors and their arrangements and installation. The Moment Tensor Inversion (MTI) was gradually designed to compute mining persuaded seismicity statistics, which is now profoundly used in the laboratory environment for seismology to observe structural material structure. It is highly focused on aspects of the MTI toolbox scheme that can record data and execute the inversion of Acoustic Emission (AE). This arrangement results in finding the next moment tensor inspection and least-squares process for S- and P-waves. Then, this 3D network is differentiated theoretically from a 2D versioned network [136]. Nassif et al. [137] compare the dynamic live load tests with a non-contact laser Doppler vibrometer (LDV) system with two contact sensors. The LDV measures girder deflections and vibrations, while the LVDT and geophone sensors measure velocity. Field tests on the Doremus Avenue Bridge Replacement Project indicate that LDV measurements are better than contact sensors and can be a replacement. Table 1 represents various sensors frequently used for SHM and their applications.

### 3.14. General Operations of Sensors and Data Acquisition in SHM

A sensor is a device that can sense/detect physical quantities such as force, stress, strains, and temperature and then convert them to an expected outcome, such as an electrical signal, to measure the applied quantitative measurements. A sensor alone was insufficient to evaluate the data received. In these situations, a signal conditioning unit keeps the sensor’s output voltage levels within the required range concerning the end device [138,139,140]. Strain, displacement, acceleration, temperature, fibre optic, and MEMS sensors have all been utilised in the SHM. Sensing characteristics such as temperature, deformation, deformation detection using fibre optics, and other factors are included in the sensing techniques. Sensors are employed in passive and active control, allowing for online evaluation, fast evaluation during accidents, and lower inspection costs. Strain sensors are often utilised because they have a longer lifespan.

Sensor data collection is critical for collecting information from sensors and processing it for use in various applications [141,142]. SHM processes data from multiple sensors and data collection units to track structures. The voltage signals from the sensors are typically translated into digital data, which the computer system reads using. DAQ can be adopted for both wireless and wired sensors. Appendix A displays some of the essential smart sensors used in SHM.

## 4. Comparative Analysis and Recommendations

### 4.1. Comparative Studies and Future Scope of Research Recommendations

This article presents an assessment of application areas and a comparative analysis of the various sensors in SHM systems [143,144]. The sensors are evaluated based on the operating mode, results obtained in multiple civil engineering applications, and compatibility with certain monitoring conditions. Based on this detailed discussion, the following comparative points and research recommendations are provided:

- SHM is typically applied in bridges with only a long-term monitoring process; therefore, the sensors and other components of SHM must be insensitive to any environmental change. Since bridge SHM is purely automated, the technician must be conversant with data processing and instrumentation to monitor all telecom activities. It is always cheaper to monitor structures for the long term than for the short term if the sensors and other related settings are appropriately designed and maintained. SHM calls for professional staff and proper installation.
- In case of failure of any of the sensors used in SHM, the absence of reading in the particular zone of the structure where the sensors were installed would be quickly realised. Therefore, sensor replacement should be carried out before catastrophic failure [145,146].
- As opposed to the accelerometer, which is more effective with the least number of sensors, other sensors require a much larger number of sensors for monitoring. Optical fibres can predict frequency levels and accelerometers; other sensors are less accurate. In accelerometers, the level of frequency and deformation may be expected with more accuracy.
- Various sensors can predict dynamic analysis based on vertical deformation and structure conditions [147]. Accelerometers may be applied for model identifications and ambient vibrations for dynamic analysis of all environmental changes.
- Accelerometers are preferable for dynamic analysis because they respond only to static parameters such as temperature, humidity, and elastic deformation. While in static loading conditions, temperature sensors, displacement transducers, and FBG have better results.
- GPS is the most accurate sensor when it comes to predicting the movement of a building. It is sensitive to small movements and is a security camera for real-time structural analysis. The GPS will sound a bell to inform people if the structure moves beyond the permitted limit.
- SHM is forecasted to predict bridges, dams, buildings, and stadiums with a compound annual growth rate of 20%. Most building firms are interested in the SHM method. SHM may be used as a compact testing and continuous monitoring system. The flexible monitoring system can monitor the current state. On the other hand, constant monitoring will acquire real-time data on a structure to identify any damage, improve sensor technologies for identifying structural alterations, and enhance wireless communication technology to enhance the effectiveness of SHM at a cheaper price [148,149].
- In SHM, a multilevel system can integrate global and local diagnostics. Diagnostics will fast condition screening worldwide, and diagnostics will fast location and degree of damage nearby. For an accurate prognosis of the health of any structure, SHM requires a deeper understanding of the equipment, signals, and mathematical techniques. They are designing low-cost dense sensor arrays and techniques for powering the sensing system by utilising energy from the structure’s working environment.

The paper also contrasts the kind of sensors applied to civil engineering and the future of SHM in the business. Regarding the performance study, it could not be refuted that all the sensors perform well with SHM. Nevertheless, accelerometers, fibre optic sensors, and piezoelectric sensors are more popular than other sensors for better SHM practice. Further, GPS is a better sensor than others for identifying structural movement. All monitoring parameters, including cracks, indoor environment, displacement, strain, temperature, humidity, and elastic deformations, will enhance the functionality of SHM in all environments.

### 4.2. Advantages and Disadvantages of Smart Sensor Technologies in SHM

New smart sensor technologies are disruptive in SHM and bring significant improvements but have specific issues and constraints. The following points provide a concise overview of their advantages and disadvantages, clearly associating each feature with the respective sensor type [150]:

- Fibre optic sensors (FOS) and piezoelectric sensors allow for the identification of structural changes and their constant monitoring for a long time. However, managing sensor networks over long periods can be expensive and requires professionalism.
- Accelerometers provide dynamic measurements, while displacement measurements are as accurate as GPS sensors. Nevertheless, GPS systems are sensitive to signal interferences, while accelerometers can be less efficient in static observation.
- MEMS and FOS are wireless sensors that allow remote health monitoring, thus enhancing safety and productivity. However, ensuring dependable wireless connections in areas with low or no easy access or where there are physical constraints, such as restricted access to power sources, can be a real problem.
- MEMS and EMI sensors, smart sensors, and analytics for condition-based monitoring help lower repair costs. However, the large volumes of data generated require efficient storage, management and analysis systems.
- MEMS and piezoelectric sensors are small, inexpensive, and ideal for small-scale applications, while integrating large-scale SHM systems involves substantial initial investments in sensors, communication networks, and calibration.
- FOS and piezoelectric sensors, for example, must endure temperature changes, corrosion and mechanical stress. Durability is still essential to sustaining the overall sensor performance and the quality of the data collected.
- The integration of smart sensor technologies in HMS, leveraging IoT-enabled systems enhances energy performance visualisation, building occupancy tracking, and post-occupancy energy analysis. With sensors, the buildings demonstrate how advanced monitoring solutions, combined with BIM, can support sustainable operations and maintenance while achieving significant environmental benefits [151].

Smart sensor integration has limitations, such as a large amount of data, complicated systems and sometimes harsh environments that affect the sensors. Maintaining cost-effective deployment, dependable communication, and sensor calibration are essential to improve SHM performance. Miniaturisation, wireless connectivity, self-diagnostic features, and AI and IoT integration will enhance the operation of SHM systems. These innovations will allow for predictive modelling, real-time decision-making and cost-effective, scalable monitoring solutions for sustainable infrastructure management.

## 5. Conclusions

The article explores various intelligent sensors employed in civil engineering practises, mainly focusing on operational applications in SHM for diverse structures like buildings, bridges, dams, and structural elements. Drawing insights from high-quality literature, the study elucidates key strategies and advancements in civil engineering and sensor technologies, presenting the main findings derived from comprehensive research. Critical sensors such as fibre optic sensors (FOS), Micro-Electro-Mechanical Systems (MEMS), piezoelectric (PZT), acceleration, displacement, strain, linear variable differential transformers (LVDT), Global Positioning System (GPS), and temperature sensors are examined, along with their application techniques. Fibre optic and piezoelectric sensors emerge as prominent tools in SHM, offering extensive global and local monitoring capabilities, with a detailed exploration of their potential for predicting damages. The study underscores the importance of SHM in civil engineering, illustrating its necessity through examples drawn from the primary literature and showcasing various fibre optic sensor applications for monitoring structural characteristics.

Moreover, the significance of piezoceramic sensors in studying building conditions and using sensors for acceleration measurement, including force, piezoelectric, and MEMS sensors, are discussed to aid in impact load prediction and damage assessment. Additionally, the paper addresses the utilisation of GPS and LVDT sensors for displacement measurements, highlighting their role in static and dynamic analysis. The discussion concludes with an overview of SHM’s capacity to anticipate early-stage damage, examining different sensors’ applications for forecasting civil constructions and summarising the predictive capabilities of SHM through static and dynamic analysis. Ultimately, the paper underscores the importance of sensor inputs in ensuring structural safety and reliability, emphasising SHM’s role in elucidating the actual state of structures.

## Figures and Tables

**Table 1:** Primary sensors used for SHM and its application.

S. No.	Primary Sensors Used in SHM	Measured Parameters	Types of Structures Adopted These Sensors, Particularly
1	Fibre optic sensor	Strain Temperature Displacement Pressure	Long pipeline work in the oil and gas sector Bridges
2	Piezoelectric sensors	Dynamic behaviour Structural stiffness Displacement Strain	Structural elements Spot welded joints Bridges
3	MEMS accelerometers	Vibration sensing Stress Natural frequency Damping ratios Mode shapes	Heritage buildings Residential buildings Bridges
4	Global positioning satellites	Acceleration Fluctuation Dynamic displacement	Bridges Dams Tower structures
5	Linear variable differential transformer	Deflection Crack monitoring Movement in joints Fluctuation	Bridge Dams Buildings

## Data Availability

Not applicable.

## Appendix A

**Table A1:** Some of the essential smart sensors used in SHM.

Sensor Name	Acronym	Description
Fibre Optic Sensors	FOS	Measures strain, temperature, vibration, and displacement; highly accurate.
Piezoelectric Sensors	PZT	Converts mechanical stress into electrical signals for vibration monitoring.
Micro-Electro-Mechanical Systems	MEMS	Compact sensors that measure vibrations, accelerations, and strains.
Global Positioning System	GPS	Tracks precise displacements and movements in civil structures.
Linear Variable Differential Transformer	LVDT	Measures small linear displacements with high resolution and reliability.
Electromechanical Impedance Techniques	EMI	Detects structural damage through impedance variations in localised regions.
Doppler Effect Sensors	-	Measures velocity by analysing frequency shifts in reflected waves.
Accelerometers	-	Monitors dynamic forces, accelerations, and vibrations in civil structures.

## References

[1] Girotto C.D., Piadeh F., Bkhtiari V., Behzadian K., Chen A.S., Campos L.C., Zolgharni M. (2024). A Critical Review of Digital Technology Innovations for Early Warning of Water-Related Disease Outbreaks Associated with Climatic Hazards. Int. J. Disaster Risk Reduct..

[2] Broer A.A.R., Benedictus R., Zarouchas D. (2022). The Need for Multi-Sensor Data Fusion in Structural Health Monitoring of Composite Aircraft Structures. Aerospace.

[3] Innes M., Davis C., Rosalie C., Norman P., Rajic N. (2017). Acoustic Emission Detection and Characterisation Using Networked FBG Sensors. Procedia Eng..

[4] Sasy Chan Y.W., Zhou Z. (2014). Advances of FRP-Based Smart Components and Structures. Pac. Sci. Rev..

[5] Spencer B.F., Ruiz-Sandoval M.E., Kurata N. (2004). Smart Sensing Technology: Opportunities and Challenges. Struct. Control Health Monit..

[6] Ručevskis S., Rogala T., Katunin A. (2022). Optimal Sensor Placement for Modal-Based Health Monitoring of a Composite Structure. Sensors.

[7] Tennyson R.C., Mufti A.A., Rizkalla S., Tadros G., Benmokrane B. (2001). Structural Health Monitoring of Innovative Bridges in Canada with Fiber Optic Sensors. Smart Mater. Struct..

[8] Cho S., Park J., Jung H.J., Yun C.B., Jang S., Jo H., Spencer B.F., Nagayama T., Seo J.W. (2010). Structural Health Monitoring of a Cable-Stayed Bridge Using Acceleration Data via Wireless Smart Sensor Network. Bridge Maintenance, Safety, Management and Life-Cycle Optimization, Proceedings of the 5th International Conference on Bridge Maintenance, Safety and Management, Philadelphia, PA, USA, 11–15 July 2010.

[9] Wu T., Liu G., Fu S., Xing F. (2020). Recent Progress of Fiber-Optic Sensors for the Structural Health Monitoring of Civil Infrastructure. Sensors.

[10] Bado M.F., Casas J.R. (2021). A Review of Recent Distributed Optical Fiber Sensors Applications for Civil Engineering Structural Health Monitoring. Sensors.

[11] Li H., Ou J. (2011). Structural Health Monitoring: From Sensing Technology Stepping to Health Diagnosis. Procedia Eng..

[12] Wright R.F., Lu P., Devkota J., Lu F., Ziomek-Moroz M., Ohodnicki P.R. (2019). Corrosion Sensors for Structural Health Monitoring of Oil and Natural Gas Infrastructure: A Review. Sensors.

[13] Sun Y., Yan Y., Tian S., Liu G., Wu F., Wang P., Gao M. (2024). Wireless Sensing in High-Speed Railway Turnouts with Battery-Free Materials and Devices. iScience.

[14] Sonbul O.S., Rashid M. (2023). Towards the Structural Health Monitoring of Bridges Using Wireless Sensor Networks: A Systematic Study. Sensors.

[15] Ferreira P.M., Machado M.A., Carvalho M.S., Vidal C. (2022). Embedded Sensors for Structural Health Monitoring: Methodologies and Applications Review. Sensors.

[16] Al-Ali A.R., Beheiry S., Alnabulsi A., Obaid S., Mansoor N., Odeh N., Mostafa A. (2024). An IoT-Based Road Bridge Health Monitoring and Warning System. Sensors.

[17] Shaharuddin S., Abdul Maulud K.N., Syed Abdul Rahman S.A.F., Che Ani A.I., Pradhan B. (2023). The Role of IoT Sensor in Smart Building Context for Indoor Fire Hazard Scenario: A Systematic Review of Interdisciplinary Articles. Internet Things.

[18] Yan S., Ma H., Li P., Song G., Wu J. (2017). Development and Application of a Structural Health Monitoring System Based on Wireless Smart Aggregates. Sensors.

[19] Zhang Y., Bai L. (2015). Rapid Structural Condition Assessment Using Radio Frequency Identification (RFID) Based Wireless Strain Sensor. Autom. Constr..

[20] Almuhammadi K., Yudhanto A., Lubineau G. (2019). Real-Time Electrical Impedance Monitoring of Carbon Fiber-Reinforced Polymer Laminates Undergoing Quasi-Static Indentation. Compos. Struct..

[21] Chacón R., Zorrilla R. (2015). Structural Health Monitoring in Incrementally Launched Steel Bridges: Patch Loading Phenomena Modeling. Autom. Constr..

[22] Deng W., Mou Y., Kashiwa T., Escalera S., Nagai K., Nakayama K., Matsuo Y., Prendinger H. (2020). Vision Based Pixel-Level Bridge Structural Damage Detection Using a Link ASPP Network. Autom. Constr..

[23] Dong W., Huang Y., Lehane B., Ma G. (2020). XGBoost Algorithm-Based Prediction of Concrete Electrical Resistivity for Structural Health Monitoring. Autom. Constr..

[24] Lin F., Scherer R.J. (2020). Concrete Bridge Damage Detection Using Parallel Simulation. Autom. Constr..

[25] Ahmed S., Thostenson E.T., Schumacher T., Doshi S.M., McConnell J.R. (2018). Integration of Carbon Nanotube Sensing Skins and Carbon Fiber Composites for Monitoring and Structural Repair of Fatigue Cracked Metal Structures. Compos. Struct..

[26] Zhu J., Wang Y., Qing X. (2019). Modified Electromechanical Impedance-Based Disbond Monitoring for Honeycomb Sandwich Composite Structure. Compos. Struct..

[27] McKenzie I., Jones R., Marshall I.H., Galea S. (2000). Optical Fibre Sensors for Health Monitoring of Bonded Repair Systems. Compos. Struct..

[28] Wang Y., Wang Y., Wan B., Han B., Cai G., Li Z. (2018). Properties and Mechanisms of Self-Sensing Carbon Nanofibers/Epoxy Composites for Structural Health Monitoring. Compos. Struct..

[29] Nanukuttan S., Yang K., Basheer P.A.M. (2023). Non-Destructive Testing and Structural Health Monitoring. ICE Handbook of Concrete Durability: A Practical Guide to the Design of Resilient Concrete Structures.

[30] Preethichandra D.M.G., Suntharavadivel T.G., Kalutara P., Piyathilaka L., Izhar U. (2023). Influence of Smart Sensors on Structural Health Monitoring Systems and Future Asset Management Practices. Sensors.

[31] Pregnolato M., Gunner S., Voyagaki E., De Risi R., Carhart N., Gavriel G., Tully P., Tryfonas T., Macdonald J., Taylor C. (2022). Towards Civil Engineering 4.0: Concept, Workflow and Application of Digital Twins for Existing Infrastructure. Autom. Constr..

[32] Gardiner P.T. (1992). Smart Structures and Materials Systems. IFAC Proc. Vol..

[33] Kasprzyk-Hordern B., Adams B., Adewale I.D., Agunbiade F.O., Akinyemi M.I., Archer E., Badru F.A., Barnett J., Bishop I.J., Di Lorenzo M. (2022). Wastewater-Based Epidemiology in Hazard Forecasting and Early-Warning Systems for Global Health Risks. Environ. Int..

[34] Hassani S., Dackermann U. (2023). A Systematic Review of Optimization Algorithms for Structural Health Monitoring and Optimal Sensor Placement. Sensors.

[35] Spencer B.F., Park J.W., Mechitov K.A., Jo H., Agha G. (2017). Next Generation Wireless Smart Sensors Toward Sustainable Civil Infrastructure. Procedia Eng..

[36] Di Graziano A., Marchetta V., Cafiso S. (2020). Structural Health Monitoring of Asphalt Pavements Using Smart Sensor Networks: A Comprehensive Review. J. Traffic Transp. Eng. (Engl. Ed.).

[37] Adeyeye K. (2023). The Householder Is King: Engendering Householder Participation in Bridging the Performance Gap in Homes. Energy Res. Soc. Sci..

[38] Ji S.H., Cho J.H., Paik J.H., Yun J., Yun J.S. (2017). Poling Effects on the Performance of a Lead-Free Piezoelectric Nanofiber in a Structural Health Monitoring Sensor. Sens. Actuators A Phys..

[39] Huang M., Huang M., Zhang J., Li J., Deng Z., Luo J. (2024). Damage identification of steel bridge based on data augmentation and adaptive optimization neural network. Struct. Health Monit..

[40] López-Castro B., Haro-Baez A.G., Arcos-Aviles D., Barreno-Riera M., Landázuri-Avilés B. (2022). A Systematic Review of Structural Health Monitoring Systems to Strengthen Post-Earthquake Assessment Procedures. Sensors.

[41] Mustapha S., Lu Y., Ng C.T., Malinowski P. (2021). Sensor Networks for Structures Health Monitoring: Placement, Implementations, and Challenges—A Review. Vibration.

[42] Jia J., Li Y. (2023). Deep Learning for Structural Health Monitoring: Data, Algorithms, Applications, Challenges, and Trends. Sensors.

[43] Liu G., Wang Q.A., Jiao G., Dang P., Nie G., Liu Z., Sun J. (2023). Review of Wireless RFID Strain Sensing Technology in Structural Health Monitoring. Sensors.

[44] Chang P.C., Flatau A., Liu S.C. (2003). Review Paper: Health Monitoring of Civil Infrastructure. Struct. Health Monit..

[45] Sbarufatti C., Manes A., Giglio M. (2014). Application of Sensor Technologies for Local and Distributed Structural Health Monitoring. Struct. Control Health Monit..

[46] Ren W., Zhang Y., Liang W.Y., Yang X.P., Jiang W.D., Liu X.H., Zhang W. (2021). A Facile and Sensitive Ratiometric Fluorescence Sensor for Rapid Visual Monitoring of Trace Resorcinol. Sens. Actuators B Chem..

[47] Hegedűs G., Czigány T. (2018). Analysis of the Applicability of Optical Fibers as Sensors for the Structural Health Monitoring of Polymer Composites: The Relationship between Attenuation and the Deformation of the Fiber. Sens. Actuators A Phys..

[48] Yang C., Oyadiji S.O. (2016). Development of Two-Layer Multiple Transmitter Fibre Optic Bundle Displacement Sensor and Application in Structural Health Monitoring. Sens. Actuators A Phys..

[49] Biondi L., Perry M., McAlorum J., Vlachakis C., Hamilton A. (2020). Geopolymer-Based Moisture Sensors for Reinforced Concrete Health Monitoring. Sens. Actuators B Chem..

[50] López-Higuera J.M., Cobo L.R., Incera A.Q., Cobo A. (2011). Fiber Optic Sensors in Structural Health Monitoring. J. Light. Technol..

[51] Curran K., Doherty G., O’Callaghan D. (2009). Wireless Sensor Networks. Understanding the Internet: A Glimpse into the Building Blocks, Applications, Security and Hidden Secrets of the Web.

[52] Zheng Y., Zhu Z.W., Xiao W., Deng Q.X. (2020). Review of Fiber Optic Sensors in Geotechnical Health Monitoring. Opt. Fiber Technol..

[53] Sun M., Staszewski W.J., Swamy R.N. (2010). Smart Sensing Technologies for Structural Health Monitoring of Civil Engineering Structures. Adv. Civil. Eng..

[54] Gianti M.S., Prasetyo E., Wijaya A.D., Berliandika S., Marzuki A. (2017). Vibration Measurement of Mathematical Pendulum Based on Macrobending-Fiber Optic Sensor as a Model of Bridge Structural Health Monitoring. Procedia Eng..

[55] Du C., Dutta S., Kurup P., Yu T., Wang X. (2020). A Review of Railway Infrastructure Monitoring Using Fiber Optic Sensors. Sens. Actuators A Phys..

[56] Sasi D., Philip S., David R., Swathi J. (2020). A Review on Structural Health Monitoring of Railroad Track Structures Using Fiber Optic Sensors. Mater. Today Proc..

[57] Irfan M.S., Khan T., Hussain T., Liao K., Umer R. (2021). Carbon Coated Piezoresistive Fiber Sensors: From Process Monitoring to Structural Health Monitoring of Composites—A Review. Compos. Part. A Appl. Sci. Manuf..

[58] Liao W., Zhuang Y., Zeng C., Deng W., Huang J., Ma H. (2020). Fiber Optic Sensors Enabled Monitoring of Thermal Curling of Concrete Pavement Slab: Temperature, Strain and Inclination. Measurement.

[59] Floris I., Adam J.M., Calderón P.A., Sales S. (2021). Fiber Optic Shape Sensors: A Comprehensive Review. Opt. Lasers Eng..

[60] Bremer K., Wollweber M., Weigand F., Rahlves M., Kuhne M., Helbig R., Roth B. (2016). Fibre Optic Sensors for the Structural Health Monitoring of Building Structures. Procedia Technol..

[61] Fan L., Tan X., Zhang Q., Meng W., Chen G., Bao Y. (2020). Monitoring Corrosion of Steel Bars in Reinforced Concrete Based on Helix Strains Measured from a Distributed Fiber Optic Sensor. Eng. Struct..

[62] Nagayama T., Sim S.H., Miyamori Y., Spencer B.F. (2007). Issues in Structural Health Monitoring Employing Smart Sensors. Smart Struct. Syst..

[63] Navabian N., Beskhyroun S., Matulich J. (2022). Development of Wireless Smart Sensor Network for Vibration-Based Structural Health Monitoring of Civil Structures. Struct. Infrastruct. Eng..

[64] Perera R., Torres L., Ruiz A., Barris C., Baena M. (2019). An Emi-Based Clustering for Structural Health Monitoring of NSM FRP Strengthening Systems. Sensors.

[65] Amezquita-Sanchez J.P., Valtierra-Rodriguez M., Adeli H. (2018). Wireless Smart Sensors for Monitoring the Health Condition of Civil Infrastructure. Sci. Iran..

[66] Bhalla S., Kaur N. (2018). Prognosis of Low-Strain Fatigue Induced Damage in Reinforced Concrete Structures Using Embedded Piezo-Transducers. Int. J. Fatigue.

[67] Hasni H., Alavi A.H., Lajnef N., Abdelbarr M., Masri S.F., Chakrabartty S. (2017). Self-Powered Piezo-Floating-Gate Sensors for Health Monitoring of Steel Plates. Eng. Struct..

[68] Moharana S., Bhalla S. (2014). A Continuum Based Modelling Approach for Adhesively Bonded Piezo-Transducers for EMI Technique. Int. J. Solids Struct..

[69] Zhang M., Bareille O., Salvia M. (2019). Cure and Damage Monitoring of Flax Fiber-Reinforced Epoxy Composite Repairs for Civil Engineering Structures Using Embedded Piezo Micro-Patches. Constr. Build. Mater..

[70] Haq M., Bhalla S., Naqvi T. (2020). Fatigue Damage and Residual Fatigue Life Assessment in Reinforced Concrete Frames Using PZT-Impedance Transducers. Cem. Concr. Compos..

[71] Haq M., Bhalla S., Naqvi T. (2020). Fatigue Damage Monitoring of Reinforced Concrete Frames Using Wavelet Transform Energy of PZT-Based Admittance Signals. Measurement.

[72] Pan H.H., Huang M.W. (2020). Piezoelectric Cement Sensor-Based Electromechanical Impedance Technique for the Strength Monitoring of Cement Mortar. Constr. Build. Mater..

[73] Xu D., Banerjee S., Wang Y., Huang S., Cheng X. (2015). Temperature and Loading Effects of Embedded Smart Piezoelectric Sensor for Health Monitoring of Concrete Structures. Constr. Build. Mater..

[74] Ahmadi J., Feirahi M.H., Farahmand-Tabar S., Keshvari Fard A.H. (2020). A Novel Approach for Non-Destructive EMI-Based Corrosion Monitoring of Concrete-Embedded Reinforcements Using Multi-Orientation Piezoelectric Sensors. Constr. Build. Mater..

[75] Jiang S.F., Wang J., Tong S.Y., Ma S.L., Tuo M.B., Li W.J. (2021). Damage Monitoring of Concrete Laminated Interface Using Piezoelectric-Based Smart Aggregate. Eng. Struct..

[76] Lezgy-Nazargah M., Saeidi-Aminabadi S., Yousefzadeh M.A. (2019). Design and Fabrication of a New Fiber-Cement-Piezoelectric Composite Sensor for Measurement of Inner Stress in Concrete Structures. Arch. Civ. Mech. Eng..

[77] Tibaduiza Burgos D.A., Gomez Vargas R.C., Pedraza C., Agis D., Pozo F. (2020). Damage Identification in Structural Health Monitoring: A Brief Review from Its Implementation to the Use of Data-Driven Applications. Sensors.

[78] Das S., Saha P. (2018). A Review of Some Advanced Sensors Used for Health Diagnosis of Civil Engineering Structures. Measurement.

[79] Lynch J.P. (2006). A Summary Review of Wireless Sensors and Sensor Networks for Structural Health Monitoring. Shock Vib. Dig..

[80] Rice J.A., Mechitov K., Sim S.H., Nagayama T., Jang S., Kim R., Spencer B.F., Agha G., Fujino Y. (2010). Flexible Smart Sensor Framework for Autonomous Structural Health Monitoring. Smart Struct. Syst..

[81] Sabato A., Dabetwar S., Kulkarni N.N., Fortino G. (2023). Noncontact Sensing Techniques for AI-Aided Structural Health Monitoring: A Systematic Review. IEEE Sens. J..

[82] Ye X.W., Jin T., Yun C.B. (2019). A Review on Deep Learning-Based Structural Health Monitoring of Civil Infrastructures. Smart Struct. Syst..

[83] Sivagami A., Jayakumar S., Kandavalli M.A. (2020). Structural Health Monitoring Using Smart Sensors. AIP Conference Proceedings.

[84] Annamdas V.G.M., Bhalla S., Soh C.K. (2017). Applications of Structural Health Monitoring Technology in Asia. Struct. Health Monit..

[85] Hassani S., Mousavi M., Gandomi A.H. (2022). Structural Health Monitoring in Composite Structures: A Comprehensive Review. Sensors.

[86] Harms T., Sedigh S., Bastianini F. (2010). Structural Health Monitoring of Bridges Using Wireless Sensor Networks. IEEE Instrum. Meas. Mag..

[87] Sreevallabhan K., Chand B.N., Ramasamy S. (2017). Structural Health Monitoring Using Wireless Sensor Networks. IOP Conf. Ser. Mater. Sci. Eng..

[88] Li H.N., Ren L., Jia Z.G., Yi T.H., Li D.S. (2016). State-of-the-Art in Structural Health Monitoring of Large and Complex Civil Infrastructures. J. Civ. Struct. Health Monit..

[89] Rice J.A., Mechitov K.A., Sim S.H., Spencer B.F., Agha G.A. (2011). Enabling Framework for Structural Health Monitoring Using Smart Sensors. Struct. Control Health Monit..

[90] Spencer B.F., Jo H., Mechitov K.A., Li J., Sim S.H., Kim R.E., Cho S., Linderman L.E., Moinzadeh P., Giles R.K. (2016). Recent Advances in Wireless Smart Sensors for Multi-Scale Monitoring and Control of Civil Infrastructure. J. Civ. Struct. Health Monit..

[91] Sreenath S., Malik H., Husnu N., Kalaichelavan K. (2020). Assessment and Use of Unmanned Aerial Vehicle for Civil Structural Health Monitoring. Procedia Comput. Sci..

[92] Lin C., Zhang C.L., Chen J.H. (2020). Optimal Arrangement of Structural Sensors in Soft Rock Tunnels Based Industrial IoT Applications. Comput. Commun..

[93] Das S., Saha P. (2021). Performance of Swarm Intelligence Based Chaotic Meta-Heuristic Algorithms in Civil Structural Health Monitoring. Measurement.

[94] Roopa A.K., Hunashyal A.M., Venkaraddiyavar P., Ganachari S.V. (2020). Smart Hybrid Nano Composite Concrete Embedded Sensors for Structural Health Monitoring. Mater. Today Proc..

[95] Ayyildiz C., Erdem H.E., Dirikgil T., Dugenci O., Kocak T., Altun F., Gungor V.C. (2019). Structure Health Monitoring Using Wireless Sensor Networks on Structural Elements. Ad Hoc Netw..

[96] Sazonov E., Janoyan K., Jha R. (2004). Wireless Intelligent Sensor Network for Autonomous Structural Health Monitoring. Smart Structures and Materials 2004: Smart Sensor Technology and Measurement Systems.

[97] Abdulkarem M., Samsudin K., Rokhani F.Z., A Rasid M.F. (2020). Wireless Sensor Network for Structural Health Monitoring: A Contemporary Review of Technologies, Challenges, and Future Direction. Struct. Health Monit..

[98] Sofi A., Jane Regita J., Rane B., Lau H.H. (2022). Structural Health Monitoring Using Wireless Smart Sensor Network—An Overview. Mech. Syst. Signal Process.

[99] Ferri Aliabadi M.H., Khodaei Z.S. (2017). Structural Health Monitoring for Advanced Composite Structures.

[100] Barthorpe R.J., Worden K. (2020). Emerging Trends in Optimal Structural Health Monitoring System Design: From Sensor Placement to System Evaluation. J. Sens. Actuator Netw..

[101] Al-Zuriqat T., Chillón Geck C., Dragos K., Smarsly K. (2023). Adaptive Fault Diagnosis for Simultaneous Sensor Faults in Structural Health Monitoring Systems. Infrastructures.

[102] Tondolo F., Cesetti A., Matta E., Quattrone A., Sabia D. (2018). Smart Reinforcement Steel Bars with Low-Cost MEMS Sensors for the Structural Health Monitoring of RC Structures. Constr. Build. Mater..

[103] Guidorzi R., Diversi R., Vincenzi L., Mazzotti C., Simioli V. (2014). Structural Monitoring of a Tower by Means of MEMS-Based Sensing and Enhanced Autoregressive Models. Eur. J. Control.

[104] Kabir M., Kazari H., Ozevin D. (2018). Piezoelectric MEMS Acoustic Emission Sensors. Sens. Actuators A Phys..

[105] Heo G., Jeon J. (2017). A Study on the Data Compression Technology-Based Intelligent Data Acquisition (IDAQ) System for Structural Health Monitoring of Civil Structures. Sensors.

[106] Bremer K., Weigand F., Zheng Y., Alwis L.S., Helbig R., Roth B. (2017). Structural Health Monitoring Using Textile Reinforcement Structures with Integrated Optical Fiber Sensors. Sensors.

[107] Bednarska K., Sobotka P., Woliński T.R., Zakrecka O., Pomianek W., Nocoń A., Lesiak P. (2020). Hybrid Fiber Optic Sensor Systems in Structural Health Monitoring in Aircraft Structures. Materials.

[108] Kim K., Choi J., Chung J., Koo G., Bae I.H., Sohn H. (2018). Structural Displacement Estimation through Multi-Rate Fusion of Accelerometer and RTK-GPS Displacement and Velocity Measurements. Measurement.

[109] Vazquez B. G.E., Gaxiola-Camacho J.R., Bennett R., Guzman-Acevedo G.M., Gaxiola-Camacho I.E. (2017). Structural Evaluation of Dynamic and Semi-Static Displacements of the Juarez Bridge Using GPS Technology. Measurement.

[110] Di Sante R. (2015). Fibre Optic Sensors for Structural Health Monitoring of Aircraft Composite Structures: Recent Advances and Applications. Sensors.

[111] Chen Z., Zhou X., Wang X., Dong L., Qian Y. (2017). Deployment of a Smart Structural Health Monitoring System for Long-Span Arch Bridges: A Review and a Case Study. Sensors.

[112] D’Alessandro A., Birgin H.B., Cerni G., Ubertini F. (2022). Smart Infrastructure Monitoring through Self-Sensing Composite Sensors and Systems: A Study on Smart Concrete Sensors with Varying Carbon-Based Filler. Infrastructures.

[113] Bedon C., Bergamo E., Izzi M., Noè S. (2018). Prototyping and Validation of MEMS Accelerometers for Structural Health Monitoring—The Case Study of the Pietratagliata Cable-Stayed Bridge. J. Sens. Actuator Netw..

[114] Azhar A.S., Kudus S.A., Jamadin A., Mustaffa N.K., Sugiura K. (2024). Recent Vibration-Based Structural Health Monitoring on Steel Bridges: Systematic Literature Review. Ain Shams Eng. J..

[115] Khuc T., Nguyen T.A., Dao H., Catbas F.N. (2020). Swaying Displacement Measurement for Structural Monitoring Using Computer Vision and an Unmanned Aerial Vehicle. Measurement.

[116] Baas E.J., Riggio M., Barbosa A.R. (2021). A Methodological Approach for Structural Health Monitoring of Mass-Timber Buildings under Construction. Constr. Build. Mater..

[117] Havaran A., Mahmoudi M. (2020). Markers Tracking and Extracting Structural Vibration Utilizing Randomized Hough Transform. Autom. Constr..

[118] Chiu W.K., Ong W.H., Kuen T., Courtney F. (2017). Large Structures Monitoring Using Unmanned Aerial Vehicles. Procedia Eng..

[119] Isah B.W., Mohamad H., Ahmad N.R. (2021). Rock Stiffness Measurements Fibre Bragg Grating Sensor (FBGs) and the Effect of Cyanoacrylate and Epoxy Resin as Adhesive Materials. Ain Shams Eng. J..

[120] Morgenthal G., Eick J.F., Rau S., Taraben J. (2019). Wireless Sensor Networks Composed of Standard Microcomputers and Smartphones for Applications in Structural Health Monitoring. Sensors.

[121] Jiang S.F., Qiao Z.H., Li N.L., Luo J.B., Shen S., Wu M.H., Zhang Y. (2020). Structural Health Monitoring System Based on FBG Sensing Technique for Chinese Ancient Timber Buildings. Sensors.

[122] Kaya Y., Safak E. (2013). Real-Time Structural Health Monitoring and Damage Detection. Conference Proceedings of the Society for Experimental Mechanics Series.

[123] Huo L., Cheng H., Kong Q., Chen X. (2019). Bond-Slip Monitoring of Concrete Structures Using Smart Sensors—A Review. Sensors.

[124] Silva D.D.S., Sobrinho J.M.B., Souto C.R., Gomes R.M. (2020). Application of Electromechanical Impedance Technique in the Monitoring of Sigma Phase Embrittlement in Duplex Stainless Steel. Mater. Sci. Eng. A.

[125] Wang J., Li W., Lan C., Wei P., Luo W. (2020). Electromechanical Impedance Instrumented Piezoelectric Ring for Pipe Corrosion and Bearing Wear Monitoring: A Proof-of-Concept Study. Sens. Actuators A Phys..

[126] Li H., Ai D., Zhu H., Luo H. (2021). Integrated Electromechanical Impedance Technique with Convolutional Neural Network for Concrete Structural Damage Quantification under Varied Temperatures. Mech. Syst. Signal Process.

[127] Li H., Ai D., Zhu H., Luo H. (2019). An Orthogonal Matching Pursuit Based Signal Compression and Reconstruction Approach for Electromechanical Admittance Based Structural Health Monitoring. Mech. Syst. Signal Process.

[128] Alwis L.S.M., Bremer K., Roth B. (2021). Fiber Optic Sensors Embedded in Textile-Reinforced Concrete for Smart Structural Health Monitoring: A Review. Sensors.

[129] de Oliveira M.A., Monteiro A.V., Filho J.V. (2018). A New Structural Health Monitoring Strategy Based on PZT Sensors and Convolutional Neural Network. Sensors.

[130] Soman R., Wee J., Peters K. (2021). Optical Fiber Sensors for Ultrasonic Structural Health Monitoring: A Review. Sensors.

[131] Panah R.S., Kioumarsi M. (2021). Application of Building Information Modelling (BIM) in the Health Monitoring and Maintenance Process: A Systematic Review. Sensors.

[132] Azimi M., Eslamlou A.D., Pekcan G. (2020). Data-Driven Structural Health Monitoring and Damage Detection through Deep Learning: State-of-the-Art Review. Sensors.

[133] Dong C.Z., Ye X.W., Jin T. (2018). Identification of Structural Dynamic Characteristics Based on Machine Vision Technology. Measurement.

[134] Rothberg S.J., Allen M.S., Castellini P., Di Maio D., Dirckx J.J.J., Ewins D.J., Halkon B.J., Muyshondt P., Paone N., Ryan T. (2017). An International Review of Laser Doppler Vibrometry: Making Light Work of Vibration Measurement. Opt. Lasers Eng..

[135] Linzer L., Mhamdi L., Schumacher T. (2015). Application of a Moment Tensor Inversion Code Developed for Mining-Induced Seismicity to Fracture Monitoring of Civil Engineering Materials. J. Appl. Geophy.

[136] Vijayan D.S., Rose A.L., Arvindan S., Revathy J., Amuthadevi C. (2020). Automation Systems in Smart Buildings: A Review. J. Ambient. Intell. Humaniz. Comput..

[137] Nassif H.H., Gindy M., Davis J. (2005). Comparison of Laser Doppler Vibrometer with Contact Sensors for Monitoring Bridge Deflection and Vibration. NDT E Int..

[138] Baduge S.K., Thilakarathna S., Perera J.S., Arashpour M., Sharafi P., Teodosio B., Shringi A., Mendis P. (2022). Artificial Intelligence and Smart Vision for Building and Construction 4.0: Machine and Deep Learning Methods and Applications. Autom. Constr..

[139] Mohamad H., Beddelee A.A.A.M., Ghazali M.F., Lee H.E., Chaiyasarn K., Nasir M.Y.M. (2023). Distributed Fibre Optic Inclinometer with Cloud-Based Monitoring System. Eng. Sci. Technol. Int. J..

[140] Nakhaei M., Nakhaei P., Gheibi M., Chahkandi B., Wacławek S., Behzadian K., Chen A.S., Campos L.C. (2023). Enhancing Community Resilience in Arid Regions: A Smart Framework for Flash Flood Risk Assessment. Ecol. Indic..

[141] Krishnanunni A.V., Kaur N., Bhalla S., Singh N., Balguvhar S. (2023). Efficacy of Singly Curved Thin Piezo Transducers for Structural Health Monitoring and Energy Harvesting for RC Structures. Energy Rep..

[142] Guéguen P., Tiganescu A. (2017). Condition-Based Decision Using Traffic-Light Concept Applied to Civil Engineering Buildings. Procedia Eng..

[143] McMillan L., Fayaz J., Varga L. (2024). Domain-Informed Variational Neural Networks and Support Vector Machines Based Leakage Detection Framework to Augment Self-Healing in Water Distribution Networks. Water Res..

[144] Tiantong P., Bongkarn T., Rianyoi R., Julphunthong P. (2022). Fabrication and Characterisation of 0–3 KNLNTS Piezoelectric Ceramic/Alite Calcium Sulfoaluminate Cement Composites. J. Mater. Res. Technol..

[145] Peng Z., Li J., Hao H. (2023). Development and Experimental Verification of an IoT Sensing System for Drive-by Bridge Health Monitoring. Eng. Struct..

[146] Martín C., Garrido D., Llopis L., Rubio B., Díaz M. (2022). Facilitating the Monitoring and Management of Structural Health in Civil Infrastructures with an Edge/Fog/Cloud Architecture. Comput. Stand. Interfaces.

[147] Le T.C., Luu T.H.T., Nguyen H.P., Nguyen T.H., Ho D.D., Huynh T.C. (2022). Piezoelectric Impedance-Based Structural Health Monitoring of Wind Turbine Structures: Current Status and Future Perspectives. Energies.

[148] Ghorbani Y., Zhang S.E., Nwaila G.T., Bourdeau J.E., Safari M., Hadi Hoseinie S., Nwaila P., Ruuska J. (2023). Dry Laboratories—Mapping the Required Instrumentation and Infrastructure for Online Monitoring, Analysis, and Characterization in the Mineral Industry. Miner. Eng..

[149] Ozbek M. (2022). Smart Maintenance and Health Monitoring of Buildings and Infrastructure Using High-Resolution Laser Scanners. Buildings.

[150] Vijayan D.S., Sivasuriyan A., Devarajan P., Krejsa M., Chalecki M., Żółtowski M., Kozarzewska A., Koda E. (2023). Development of Intelligent Technologies in SHM on the Innovative Diagnosis in Civil Engineering—A Comprehensive Review. Buildings.

[151] Kurcjusz M., Mielnik A. (2024). Technology vs. creativity in architecture: Striving for synergy. Defining the Architectural Space, Architecture and Technology.

